# Cooling rates and melt extraction timescales determined by diffusion chronometry on shallow crustal plutonic rocks

**DOI:** 10.1007/s00410-025-02238-0

**Published:** 2025-07-11

**Authors:** Thomas Grocolas, Othmar Müntener, Elias M. Bloch, Stéphane Escrig, Alexey Ulyanov, Anne-Sophie Bouvier

**Affiliations:** 1 Institute of Earth Sciences, University of Lausanne, Géopolis, CH-1015 Lausanne, Switzerland; 2 Lunar and Planetary Laboratory, University of Arizona, Tucson, AZ 85721 USA; 3 Laboratory for Biological Chemistry, School of Architecture, Civil and Environmental Engineering, Ecole Polytechnique Fédérale de Lausanne, CH-1015 Lausanne, Switzerland

**Keywords:** Adamello batholith, Diffusion chronometry, Plutonic rocks, Residence time

## Abstract

Diffusion chronometry has emerged as a valuable tool to track the timescales of magmatic processes and is now routinely applied on erupted volcanic products to infer crystal residence times and mixing-to-eruption timescales. Despite some attempts to apply such a technique to plutonic rocks, slow cooling in these systems complicates the interpretation of the retrieved timescales. Here we investigate the cooling rates and crystal–melt segregation timescales in the Western Adamello and Re di Castello units of the Adamello batholith (Italy). The Western Adamello is mainly composed of tonalite and volumetrically minor cumulative gabbro and leucotonalite, and segregated granite. The studied area from the Re di Castello exhibits a concentric structure mainly composed of tonalite, granodiorite, and aplitic and pegmatitic granite. Strongly zoned plagioclase crystals occur in both leucotonalite and granodiorite samples, while quartz crystals displaying normal Ti zoning are only observed in some granodiorites. After determining the crystallisation temperatures of plagioclase mantle and rim and quartz, as well as the initial conditions prior to diffusion, cooling rates were estimated based on plagioclase mantle-to-rim and quartz profiles. The retrieved cooling paths correspond, within uncertainty, to cooling rates calculated using thermal modelling and obtained by previous studies using ^39^Ar/^40^Ar dating. Crystal–melt segregation timescales were then calculated based on the diffusion modelling of plagioclase core-to-mantle profiles and the retrieved cooling rates. The calculated timescales (~ 10^4^–10^5^ yr) likely correspond to the plagioclase core-to-mantle residence time before crystallisation of the rim. Interestingly, these crystal residence times are similar to the zircon crystallisation timespan recorded in the Western Adamello and Re di Castello units, and to the crystal residence times obtained on historical volcanic eruptions (~ 10^4^–10^6^ yr). Overall, these findings highlight that plutonic systems can be used to reconstruct magmatic timescales and support the hypothesis of a close connection between plutonism and volcanism.

**Supplementary Information:**

The online version contains supplementary material available at 10.1007/s00410-025-02238-0.

## Introduction

Almost one billion people live close to an active volcanic area. Forecasting of volcanic unrest is therefore very important and requires deep understanding of the fundamental processes leading to an eruption and their associated timescales. Volcanic eruptions are triggered by several, possibly coupled, processes including magma replenishment, volatile exsolution and buoyancy (e.g. Caricchi et al. 2014a; Degruyter and Huber 2014; Sparks et al. 1977). Reconstructing the mechanisms that operate in Earth’s crustal reservoirs is intimately linked to precisely constraining the thermal evolution of these magma bodies. Indeed, the thermal budget of crustal reservoirs exerts the main control on the physical properties of the magma, thereby regulating the eruptability of magma reservoirs. Additionally, constraining timescales associated with the assembly of eruptible magma, magma mixing prior to eruption, and melt extraction within volcanic conduits is crucial to quantify the lifespan of magma reservoirs and mitigate volcanic hazards.

The view regarding the thermal and physical conditions of magma storage has shifted toward a consensus whereby magma reservoirs are composed of a volumetrically dominant (> 50 vol.%), interconnected crystal network, hereafter referred to as “mush,” whereas crystal–poor domains (“melt”) are rare (Cashman et al. 2017; Marsh 1981). Recently, studies quantifying the thermal conditions of magma storage using zircon dating and diffusion modelling demonstrated that magma reservoirs predominantly reside at temperatures above the solidus, typically between 700 and 750 °C (e.g. Barboni et al. 2016; Cooper and Kent 2014; Tavazzani et al. 2023). In detail, this magma storage temperature is a direct function of the melt recharge rate, which is commonly divided into an instantaneous and a long-term, average magma flux (Caricchi et al. 2014a). Some studies attempted to precisely constrain the storage temperature as a function of the melt recharge rate (e.g., Caricchi et al. 2014b; Weber et al. 2020), which is critical since the investigated temperature window (700–750 °C) typically corresponds to dramatic rheologic changes within evolved magma reservoirs. Slightly peraluminous, H_2_O-rich andesitic to dacitic melts undergo a biotite-forming peritectic reaction (Grocolas and Müntener 2024), while metaluminous, less H_2_O-rich felsic melts saturate with alkali feldspar at higher temperatures (e.g. Johnson and Rutherford 1989). These two crystallisation processes substantially decrease melt fractions with a modest decrease in temperature, marking a tipping point in magma rheology. As such, precisely constraining the storage temperature of magma reservoirs and their cooling rates is essential for evaluating their eruptability, calculations of crystal residence time, and to retrieve crystal–melt segregation timescales.

Timescales associated with volcanic eruption are commonly determined using high-resolution U–Pb zircon dating (e.g. Miller et al. 2007; Walker Jr et al. 2007), U-series disequilibria (e.g. Condomines et al. 2003; Cooper and Reid 2008), crystal size distribution coupled to crystal growth rate (Marsh 1988; Randolph and Larson 1988; Higgins 2006) and diffusion chronometry (Costa et al. 2020, and references therein). The latter is now routinely applied and, by measuring elements having different diffusivities in different minerals, holds the potential to reconstruct the temporal evolution of a crustal reservoir from assembly to eruption. Most of the studies using diffusion chronometry interpret their results as rejuvenation timescales based on the presence of reverse zoning at crystal rims (e.g. Morgan et al. 2006). The calculated timescales of magma recharge prior to eruption at basaltic volcanoes generally span a few weeks to a few years, whereas decadal to millennial timescales are typical of volcanoes erupting evolved material (Cooper 2019; Costa et al. 2020). On the other hand, U-series disequilibria and zircon U–Pb systems typically yield ages ranging from 10^4^ to > 10^6^ yr, which are usually interpreted as crystal residence times (e.g. Barboni and Schoene 2014; Chambers et al. 2020; Cooper 2015; Klein et al. 2021; Samperton et al. 2015; Schaen et al. 2021).

The goal of this study is to determine timescales of melt segregation and extraction within a slowly-cooled, upper crustal plutonic body, specifically the Adamello batholith, by precisely constraining its thermal evolution. We present field relationships and detailed textural observations coupled with thermometry and multi-mineral compositional profiles to demonstrate that quartz and plagioclase rims capture the cooling history of the pluton, while plagioclase cores within in situ cumulates record residence times prior to crystal accumulation and melt segregation. We identified three zones in plagioclase recording its complex history, while quartz is only composed of two zones. Detailed thermometry using experimentally calibrated geothermometers and crystallisation experiments combined with diffusion equations were used to reproduce the diffusion profiles and calculate cooling rates and crystal–melt segregation timescales. Finally, we compare the retrieved cooling rates and timescales with a thermal model, local ^39^Ar/^40^Ar ages and high-resolution zircon U–Pb ages to benchmark the accuracy of the employed methods. Comparing our results to compiled timescale data from various systems, we emphasise that diffusion timescales obtained in plutonic rocks are comparable to zircon derived timescales and document magma reservoir differentiation and assembly of eruptible magma prior to melt extraction.

## Geological setting

**Fig. 1:**
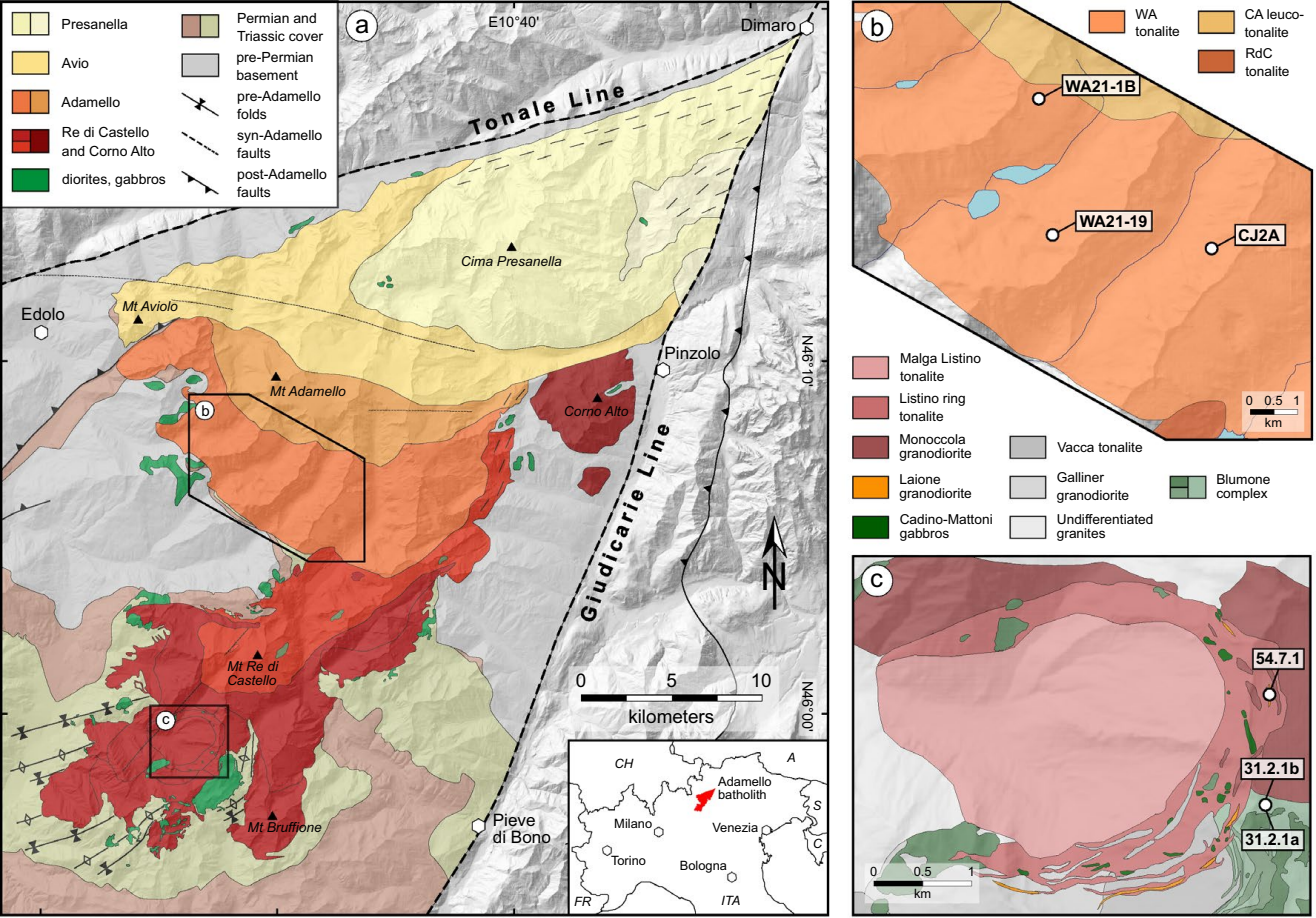
**a** Simplified geological map of the Adamello batholith exhibiting the four superunits and corresponding lithologies (modified after Schaltegger et al. 2009). Locations of field areas are also represented. **b** Geological map of the Western Adamello tonalite (modified after Floess and Baumgartner 2015) alongside sample locations. **c** Geological map of the Listino ring complex and vicinity (modified after Verberne 2013) alongside sample locations. Coordinates are reported using the WGS84 Coordinate System

The Adamello batholith, located in the Brescian Alps of northern Italy, represents the largest and oldest Paleogene calc-alkaline intrusive body in the Alps and was formed during the collision of the European and Adriatic plates (e.g. Callegari and Brack 2002). The excellently exposed Adamello pluton covers a 675 km^2^ area with up to 3 km of vertical relief and is usually separated into four superunits: Re di Castello, Adamello, Avio and Presanella (Fig. 1). These superunits are composed at 99% of quartz-diorite, tonalite and granodiorite, the remaining 1% being hornblendite and gabbro (Ulmer et al. 1983). The emplacement ages progressively decrease from South (42–38 Ma) to North (34–31 Ma) (Del Moro et al. 1983; Schaltegger et al. 2019).

The Western Adamello unit is part of the Adamello superunit (Fig. 1) and is a coarse-grained, homogeneous tonalite with large abundances of amphibole and biotite phenocrysts (~ 20 vol.%). The Western Adamello tonalite comprises an external zone of ~ 500 m at its southern border exhibiting a steeply-dipping, contact-parallel foliation interpreted as a feeder zone (Floess and Baumgartner 2015). During differentiation, the Western Adamello tonalite underwent a hornblende-consuming peritectic reaction producing biotite, and crystal–melt segregation forming cumulative hornblende-biotite-gabbro and leucotonalite (Grocolas and Müntener 2024). The extracted melt is recorded by granitic dikes. The thermal evolution of the contact aureole has been used to demonstrate that the Western Adamello tonalite was emplaced incrementally from southwest to northeast over a period of 1.2 Myr from 37.6 to 36.4 Ma at a pressure of ~ 250 MPa (Floess 2013; Floess and Baumgartner 2013).

The Listino ring complex and Lago della Vacca suite are located in the southern part of the Re di Castello superunit and form a 5-km semi-circular zone of foliated tonalites (Fig. 1) emplaced through a ballooning mechanism (John and Blundy 1993; Verberne 2013). The Monoccola granodiorite is the most outward lithology of the Listino ring complex and is a medium- to coarse-grained, homogeneous granodiorite. It contains plagioclase, amphibole, biotite and quartz phenocrysts, and interstitial alkali feldspar. A weak, inward-dipping foliation is present close to the contact with the Listino ring tonalite. The Listino ring tonalite is a medium-grained, rather homogeneous tonalite with abundant plagioclase phenocrysts. A similar magmatic inward-dipping foliation occurs and is defined by plagioclase, acicular amphibole, and biotite. The Laione granodiorite (LGD) is a hypabyssal, plagioclase-phyric granodiorite that forms small bodies (10–30 m large) included within the Listino ring tonalite displaying sharp contacts with the host tonalite (Verberne 2013). It contains alkali feldspar oikocrysts and plagioclase phenocrysts texturally similar to the plagioclase crystals from the Listino ring tonalite. In addition, the Lago della Vacca suite also contains small granodioritic bodies displaying similar sharp contacts with the host diorites. The tonalite of Malga Listino constitutes the core of the Listino ring complex and is a homogeneous, medium-grained tonalite deprived of foliation except close to the contact with the Listino ring tonalite. It texturally resembles the Monoccola granodiorite but with less modal quartz and biotite. Finally, granitic dikes (up to 1 m wide), originating from the Listino ring tonalite and Monoccola granodiorite, radially crosscut all the Listino ring complex units. These dikes are composed of plagioclase, alkali feldspar, quartz, biotite and minor garnet and display either an aplitic texture or a pegmatitic texture with aplitic rims. The high-precision U–Pb zircon ages decrease from 41.7 Myr for the outward Monoccola granodiorite to 41.2 Myr for the inward tonalite of Malga Listino (Verberne 2013).

## Methods

### Backscattered electron imaging

Zoning patterns in plagioclase were determined on carbon-coated polished thin sections using backscattered electron (BSE) images acquired with a Tescan Mira II LMU field-emission gun scanning-electron microscope (SEM) equipped with an Oxford energy dispersive chemical detector at the University of Lausanne. The acceleration voltage was set to 10 kV and the beam current between 5 and 10 nA. Changes in BSE brightness are related to differences in the mean atomic number of pixels. In plagioclase, the major component affecting the BSE brightness is the anorthite [An = 100 × molar Ca/(Ca + Na + K)] content.

### Cathodoluminescence imaging

Zoning patterns in quartz were imaged on carbon-coated polished thin sections using a cathodoluminescence (CL) detector attached to a CamScan MV2300 SEM at the University of Lausanne. The acceleration voltage was set to 10 kV and the beam current to ~ 10 nA. Initial, low-quality images containing numerous quartz crystals were first acquired so that a large number of crystals could be evaluated for their zoning patterns prior to single crystal images. Changes in luminescence in quartz have been ascribed to Ti variations (Wark and Spear 2005), but differences in Al contents can also play a role.

### Electron probe microanalysis

Mineral major element compositions were acquired on carbon-coated polished thin sections by field-emission gun electron probe microanalysis (EPMA) using a JEOL JXA-8530F HyperProbe equipped with five wavelength dispersive spectrometers (WDS) at the University of Lausanne. The acceleration voltage was set to 15 kV, the beam current to 10 nA, and the beam size ranged from 1 to 5 μm. Counting times were 30 s on the peak and 15 s on the background. Measurements were corrected with the PRZF method (Armstrong 1995) and standardised using synthetic glasses and natural minerals.

### Laser ablation inductively coupled plasma mass spectrometry (LA-ICP-MS)

Trace element composition profiles of plagioclase were determined using a Perkin Elmer NexION 5000 ICP-MS coupled with an Australian Scientific Instruments RESOlution SE 193 nm Ar-F excimer laser ablation system equipped with an S155 two-volume ablation cell at the University of Lausanne. The LA-ICP-MS system was optimised by linearly scanning the NIST SRM-612 glass standard at 10 μm s^−1^, 10 Hz repetition rate, 80 μm beam diameter, and 6 J cm^−2^ energy density in order to increase the spectrometer sensitivity (^139^La^+^  > 1.6 × 10^6^ cps) without producing significant oxides (^248^ThO^+^/^232^Th^+^  < 0.5%) and doubly-charged ions (Ba^2+^/Ba^+^  < 3.0%). Helium (1 l min^−1^) and N_2_ (1 ml min^−1^) were used as carrier gases. The data were acquired using slits to shape the laser beam as a 7.5 × 50 μm rectangular area with the long axis parallel to the diffusion interface. The raster mode was used, whereby the stage was moved at a constant speed of 1 μm s^−1^, while the other parameters were the same as for the optimisation. Background collection and wash-out times were 70 and 35 s, respectively. Dwell times were 10 ms for ^27^Al and ^42^Ca, 20 ms for ^25^ Mg, ^57^Fe, ^139^La, ^151^Eu and ^208^Pb, and 30 ms for ^49^Ti, ^86^Sr and ^137^Ba. After every five unknown measurement NIST SRM-612 and BCR-2G were measured using the raster mode. Absolute concentrations were calculated using CaO as internal reference, NIST SRM-612 as primary standard, and BCR-2G as secondary standard for quality control. Data reduction was performed with the Iolite 4 software (Paton et al. 2011). The average elemental abundances of the standards were taken from Jochum et al. (2011). The scanning depth was then checked using a white-light interferometer and ranged from 0.5 to 1 μm.

The trace element compositions of amphibole, plagioclase, alkali feldspar, quartz and zircon were acquired following the same procedure as described by Grocolas and Müntener (2024) and are reported in the Electronic Supplementary Material ESM 2. Uncertainties associated with LA-ICP-MS spot and line-scanning analysis are discussed in the Electronic Supplementary Material ESM 1.

### Secondary ion mass spectrometry

Thin sections from the Laione granodiorite were cut using a diamond wire saw and pressed in indium. The mounts were cleaned with ethanol and coated with gold (~ 35 nm) before being loaded into the sample chamber at least 24 h before analysis. Titanium concentrations in quartz from the Laione granodiorite were measured using a Cameca IMS 1280-HR secondary ion mass spectrometer (SIMS) at the University of Lausanne. The vacuum of the sample chamber was kept at ~ 6 × 10^–9^ mbar. Secondary ions of ^27^Al, ^30^Si and ^48^Ti produced by a primary O^−^ beam of ~ 3 nA were measured using the axial electron multiplier for ^27^Al and ^48^Ti, and a Faraday cup (FC2) for ^30^Si. The field aperture was set at 5,000 μm and the energy window at 50 eV. The analysed area was pre-sputtered for 1 min followed by 6 min of data collection over an area of ~ 6 × 6 μm. Entrance (~ 80 μm) and exit (~ 400 μm) slits were adjusted to get a mass resolving power (MRP) of ~ 3,000. Secondary ^48^Ti ion intensities were normalised to ^30^Si and quantified using one natural quartz standard with known Ti concentrations (Audétat et al. 2015). Uncertainties associated with SIMS analysis are discussed in the Electronic Supplementary Material ESM 1.

### Nanoscale secondary ion mass spectrometry (NanoSIMS)

High-resolution profiles of Ti in quartz and Sr in plagioclase for the Laione granodiorite and Western Adamello leucotonalite, respectively, were determined using a Cameca NanoSIMS 50L at the University of Lausanne. Areas of interest were sputtered with a primary ^16^O^−^ beam focused to a spot size of ~ 800 nm. Following an implantation phase required to reach a significant emission of secondary ions, profile locations were defined perpendicular to the crystal zoning. These profiles were acquired by continuously scanning the target segment for a total of 20 scans, with a dwell time of 1 and 2 s for quartz and plagioclase, respectively. Secondary ions of ^46^Ti, ^47^Ti, ^48^Ti, ^28^Si and ^29^Si for quartz, and ^27^Al, ^28^Si, ^40^Ca, ^86^Sr and ^88^Sr for plagioclase were measured using electron multipliers. The reported data correspond to the cumulated counts of the linescans free of any spikes caused by the presence of small inclusions. To remove the effect of local variation in the ionisation and extraction processes, the data are reported as ^48^Ti/^28^Si for quartz. Because of major element zoning in plagioclase, the ^86^Sr and ^88^Sr counts are not normalised. Absolute concentrations were calculated using the quartz Ti concentrations measured by SIMS coupled with the CL greyscale intensity, and the plagioclase Sr concentrations measured by LA-ICP-MS. Uncertainties associated with NanoSIMS analysis are discussed in the Electronic Supplementary Material ESM 1.

### Diffusion modelling

When a crystal exhibits a chemical gradient, Fick’s second law (Fick 1855) can be used to describe the homogenisation of this chemical gradient and calculate the thermal evolution undergone by the host crystal. This is described in one dimension by Eq. 1:

1 $$ \frac{{\delta C_{i} }}{\delta t} = D_{i}^{t} \frac{{\partial^{2} C_{i} }}{{\partial x^{2} }}, $$

where *C* is the concentration of element *i*, *t* is time, *D* is the diffusion coefficient of element *i* at time *t*, and *x* is the position along the modelled profile. This equation can be solved using the explicit finite-difference method and treated as tracer systems, whereby diffusion of each element occurs solely in response to its own concentration gradient (Crank 1975). This is described by Eq. 2 for an infinite reservoir:

2 $$ \frac{{C_{i}^{t + 1} - C_{i}^{t} }}{\Delta t} = \frac{1}{{\Delta x^{2} }}\left[ {D_{i}^{t} \left( {C_{i + 1}^{t} - 2C_{i}^{t} + C_{i - 1}^{t} } \right)} \right]. $$

By precisely determining the initial conditions, boundary conditions and diffusion coefficients, temperature–time paths were retrieved by fitting a model to the concentration gradient using MATLAB scripts developed in this study. To model cooling paths, temperature was changed at every time step and followed an exponential function, and diffusion coefficients were recalculated following Eq. 3:

3 $$ \log_{10} D_{i} = \log_{10} D_{0} - \left( {\frac{{E_{a} }}{2.303RT}} \right), $$

where log_10_*D*_0_ (m^2^ s^−1^) and *E*_a_ (J mol^−1^) are the parameters describing the diffusion of element *i*, *R* is the universal gas constant (J mol^−1^ K^−1^), and *T* is the temperature (K). The presented model assumes that crystal overgrowths have formed sufficiently fast to be treated as instantaneous compared to diffusion timescales. To observe an effect of crystal growth on diffusion, the crystal growth rate must be of the same order of magnitude as the diffusion timescale, which is unlikely for magmatic systems (e.g. Devoir et al. 2021). Uncertainties associated with diffusion modelling are discussed in the Electronic Supplementary Material ESM 1.

#### Ti in quartz

Because the initial chemical profiles are erased after the onset of diffusion, two different initial concentrations were used for diffusion modelling of Ti in quartz. The first one was a simple step-function while the second one accounts for a potential growth zone between two plateaus. The boundary conditions were fixed as diffusion did not operate further than ~ 5–10 μm. The diffusion coefficients used for Ti-in-quartz diffusion were taken from Cherniak et al. (2007), Jollands et al. (2020), and Audétat et al. (2021, 2023). A rigorous comparison between these studies is done and the retrieved timescales are discussed as a function of the diffusion coefficients.

#### Sr and Ba in plagioclase

Trace element partitioning in plagioclase is strongly dependent on temperature and plagioclase composition (Bindeman et al. 1998; Dohmen and Blundy 2014; Nielsen et al. 2017; Mutch et al. 2022), and follows an Arrhenius relationship as defined by Eq. 4:

4 $$ RT{\text{ln}}K_{{{\text{D}}_{i} }} = A_{i} \cdot X_{{{\text{An}}}} + B_{i}, $$

where *K*_D_ is the partition coefficient between plagioclase and melt for element *i*, *X*_An_ is the molar anorthite (An) content [molar Ca/(Ca + Na + K)], and both *A* and *B* are constants (J mol^−1^) and are different for each element. Likewise, Sr and Ba diffusion in plagioclase is a function of An content and temperature (Cherniak and Watson 1992, 1994; Giletti and Casserly 1994; Grocolas et al. 2025; LaTourette and Wasserburg 1998; Costa et al. 2003; Van Orman et al. 2014). Therefore, a modified solution to the diffusion equation was developed by Costa et al. (2003) to incorporate the dependence on An content (Eq. 5):

5 $$ \frac{{\partial C_{i} }}{\partial t} = D_{i}^{t} \frac{{\delta^{2} C}}{{\delta x^{2} }} - \frac{{D_{i}^{t} C_{i} }}{RT}A_{i} \frac{{\delta^{2} X_{{{\text{An}}}} }}{{\delta x^{2} }}. $$

This solution to the diffusion equation leads to a quasi-steady state profile that depends on the measured plagioclase An content, assumed to remain constant through time (Grove et al. 1984), where the chemical gradient of element *i* never reaches complete homogenisation (Costa et al. 2003; Faak et al. 2014; Dohmen et al. 2017). We then solve this equation using the explicit finite-difference method (Eq. 6):

6 $$ $$$$ \begin{aligned} \frac{{C_{i}^{t + 1} - C_{i}^{t} }}{\Delta t} & = \left[ {D_{i}^{t} \left( {\frac{{C_{i + 1}^{t} - 2C_{i}^{t} + C_{i - 1}^{t} }}{{\Delta x^{2} }}} \right)}\right.\\ & \left. \quad + \left( {\frac{{D_{i + 1}^{t} - D_{i}^{t} }}{\Delta x}} \right)\left( {\frac{{C_{i + 1}^{t} - C_{i}^{t} }}{\Delta x}} \right) \right] \\ & \quad- \frac{{A_{i} }}{RT}\left[ D_{i}^{t} \left( {\frac{{C_{i + 1}^{t} - C_{i}^{t} }}{\Delta x} \cdot \frac{{X_{{{\text{An}},i + 1}}^{t} - X_{{{\text{An}},i}}^{t} }}{\Delta x}} \right)\right.\\ & \left. \quad + C_{i}^{t} \left( {\frac{{D_{i + 1}^{t} - D_{i}^{t} }}{\Delta x} \cdot \frac{{X_{{{\text{An}},i + 1}}^{t} - X_{{{\text{An}},i}}^{t} }}{\Delta x}} \right) \right. \\ &\left. \quad + D_{i}^{t} C_{i}^{t} \left( {\frac{{X_{{{\text{An}},i + 1}}^{t} - 2X_{{{\text{An}},i}}^{t} + X_{{{\text{An}},i - 1}}^{t} }}{{\Delta x^{2} }}} \right) \right]\end{aligned}$$

Before modelling diffusion profiles, the first step involves determining the quasi-steady state profiles by fixing the rim trace element composition to the measured rim composition (Costa et al. 2003). The quasi-steady state profile is then calculated inward by using partition coefficients (Dohmen and Blundy 2014) and compared to the measured trace element profile; matching profiles occur when diffusion went to completion. In such cases, the calculated timescale and/or cooling rate only represent minimum values.

Finally, to infer initial profiles, two methods have been applied in the literature. The first one consists in using the correlation between the plagioclase An content and the trace element of interest (Druitt et al. 2012), and highly relies on the degree of diffusion of this element. It is therefore important to distinguish original magmatic trace element contents from concentrations modified by diffusion. The second method is based on the reset of the diffused profile using partitioning (Mutch et al. 2021; Lubbers et al. 2022). The observed trace element profile is divided by the partition coefficient to obtain the equilibrium melt compositions from which a simplified series of discrete melt compositions is generated. The initial trace element profile is then calculated by multiplying this simplified equilibrium melt profile with the *K*_D_. In the following, we use the first method because it is based on less assumptions, and the correlation between the plagioclase An content and trace elements has been previously evaluated for the samples investigated in this study (Grocolas and Müntener 2024). Despite being less direct, the method employed by Mutch et al. (2021) and Lubbers et al. (2022) has been demonstrated to yield the same results within uncertainty (Grocolas et al. 2025).

## Results

### Petrography

**Fig. 2:**
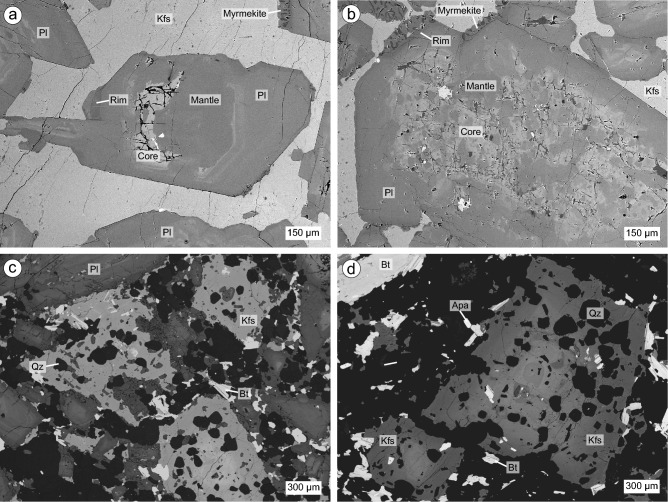
Backscatter electron images of representative crystal zoning of the studied lithologies. **a** Subhedral to euhedral plagioclase crystal from Western Adamello leucotonalite exhibiting a dissolved inner core and resorbed outer core, a homogenous mantle, and a discontinuous dark rim. Plagioclase crystals are surrounded by a single anhedral alkali feldspar oikocryst. Myrmekite occurs at some plagioclase rims (sample CJ2A; N46°05′49.7″, E10°32′16.0″). **b** Subhedral to euhedral plagioclase crystal from Western Adamello leucotonalite having a large, patchy zoned, bright core, a homogeneous mantle, and a discontinuous rim with local myrmekite. Interstitial alkali feldspar surrounds plagioclase (sample WA21-19; N46°06′26.6″, E10°29′26.8″). **c** Laione granodiorite constituted of plagioclase phenocrysts, anhedral to subhedral alkali feldspar oikocrysts with inclusions of small, subhedral quartz, biotite and plagioclase, and a fine-grained matrix composed of the same small quartz crystals, plagioclase and biotite (sample 54.7.1; N45°58′52.5″, E10°27′35.7″). **d** Laione granodiorite highlighting the presence of some biotite phenocrysts, the occurrence of apatite in the matrix assemblage, and the zoning pattern of alkali feldspar oikocrysts (sample 54.7.1; N45°58′52.5″, E10°27′35.7″)

The samples used in this study come from the Western Adamello and the Re di Castello superunits, which are part of the Adamello batholith. They consist of three leucotonalites from the Western Adamello, and three Laione granodiorites from the Listino ring and Blumone (Lago della Vacca) complexes (Fig. 1). Leucotonalites from the Western Adamello are relatively fine-grained leucocratic rocks mainly composed of plagioclase and quartz with interstitial, oikocrystic alkali feldspar, and minor amphibole and/or biotite phenocrysts (Fig. 2a, b). Field observations coupled with phase relationships and chemistry suggest that these leucotonalites represent in situ accumulations of plagioclase and quartz in non-cotectic proportions from the main tonalitic melt (Grocolas and Müntener 2024). In these rocks, plagioclase is normally zoned and can be subdivided into three main zones: (1) relatively rare calcium-rich (> An_70_), partially-resorbed, inherited cores from lower portions of the crust; (2) volumetrically dominant core-to-mantle intermediate compositions (An_40-60_); and (3) a thin (< 150 μm), sodium-rich (~ An_30_) rim that can locally be absent (Fig. 2a, b). Leucotonalite from the Western Adamello typically has a plagioclase-dominated bulk rock composition with high Sr contents (215–734 μg/g) and a wide range of positive Eu anomalies [Eu/Eu* = Eu_N_/(Sm_N_ × Gd_N_)^1/2^; 1.36–3.56] (Electronic Supplementary ESM 1).

The Laione granodiorite from the Listino ring and Blumone complexes is a porphyritic granodiorite containing large (0.5–5 mm), inclusion-free, oscillatory-zoned plagioclase phenocrysts, and large (0.5–3 mm) alkali feldspar oikocrysts (Fig. 2c, d). The latter contains inclusions of subhedral quartz crystals (50–150 μm) often forming small aggregates, small (< 100 μm) subhedral biotite, and small (< 100 μm) plagioclase crystals elongated parallel to the alkali feldspar grain boundaries. The matrix is composed of small (< 150 μm) plagioclase and biotite, and the same subhedral quartz crystals as those included in alkali feldspar. Three plagioclase zones, similar to those observed in the Western Adamello leucotonalite, are identified in the Laione granodiorite, albeit with thinner (< 30 μm) rims. Subhedral amphibole phenocrysts (1–3 mm) occur rarely. Euhedral apatite and zircon (50–250 μm) are present in the matrix or as inclusions within interstitial biotite, while rare titanite crystals (50–150 μm) are only present in the matrix. The Laione granodiorite samples from the Listino ring and Blumone complexes have homogeneous bulk rock compositions with SiO_2_ (65.7–69.6 wt.%) inversely correlated to Sr (454–375 μg/g), high Ba (630–875 μg/g) contents and no significant Eu anomaly (0.87–1.12) (Electronic Supplementary Material ESM 1). These values of Eu anomaly coupled with the compositional homogeneity of the Laione granodiorite are typical of plutonic rocks that did not undergo significant melt extraction and crystal accumulation.

### Quartz

**Fig. 3:**
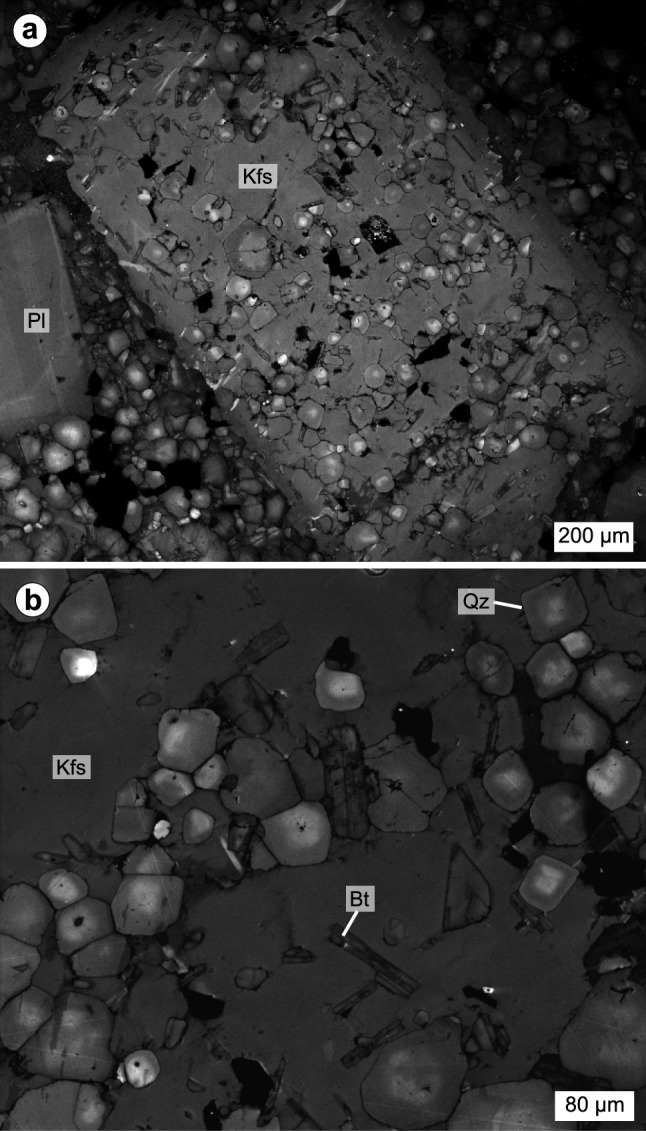
Compositional zoning observed in quartz from the Laione granodiorite. **a** Cathodoluminescence (CL) image of an alkali feldspar oikocryst highlighting the systematic normal zoning of interstitial quartz and quartz inclusions (sample 54.7.1; N45°58′52.5″, E10°27′35.7″). **b** Similar CL image focusing on the normal zoning of quartz crystals included in an alkali feldspar oikocryst (sample 54.7.1; N45°58′52.5″, E10°27′35.7″)

**Fig. 4:**
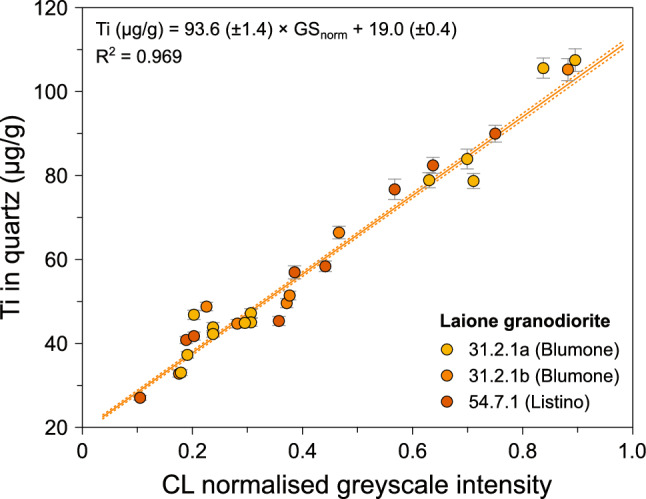
Calibration of the cathodoluminescence greyscale intensity of quartz to the Ti concentrations measured by SIMS based on quartz from three Laione granodiorite samples. The data were fit using a Monte Carlo scheme coupled to a root-mean square optimisation

Small, subhedral to euhedral quartz from the Laione granodiorite present in the matrix and as inclusions within alkali feldspar oikocrysts systematically exhibit a gradational transition from a bright core to darker rims in CL images (Fig. 3c, d). Quartz often has well-defined crystal faces, contrasting with the boundary between the crystal core and rim which is diffuse and has a rounded shape. This may arise either from (1) the growth of a bright core at emplacement pressure followed by the growth of a darker rim coupled with diffusion re-equilibration, or (2) the growth of a bright core at higher pressure followed by decompression leading to partial dissolution of the quartz core, and growth of a darker rim followed by diffusion re-equilibration. The relative brightness of quartz CL images is usually ascribed to the variations of the Ti and/or Al contents (e.g. Götze et al. 2001; Wark and Spear 2005). The small, subhedral to euhedral quartz from the Laione granodiorite have Al contents ranging from 23 to 49 μg/g with no relationship with the CL greyscale intensity. On the other hand, the quartz Ti concentrations are linked to the CL brightness, with the quartz bright cores having high Ti concentrations (79–110 μg/g) while the dark rims have lower Ti contents (28–52 μg/g). This correlation is used to calibrate the quartz Ti concentrations from the CL greyscale intensity (Fig. 4) and NanoSIMS intensity ratios.

### Plagioclase

**Fig. 5:**
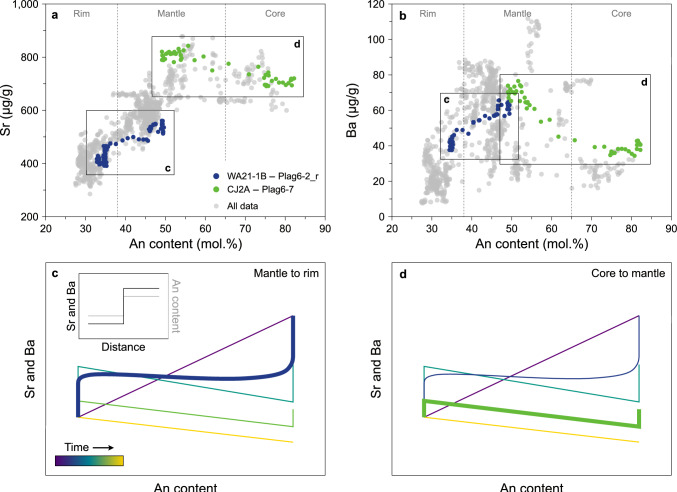
Plagioclase compositions and extent of diffusion re-equilibration. **a** Plagioclase An content (mol.%) vs. Sr (μg/g). The grey data points represent all the data measured through LA-ICP-MS line scanning, and the coloured data points highlight two representative examples of a core-to-mantle profile (sample CJ2A) and a mantle-to-rim profile (sample WA21-1B) from the Western Adamello. **b** Plagioclase An content (mol.%) vs. Ba (μg/g). The two highlighted profiles are the same as in **a**. **c** Theoretical Sr- and Ba-in-plagioclase diffusion model for a mantle-to-rim profile plotted as plagioclase An content vs. trace elements. The inset represents the initial conditions for the trace elements and An content as a function of the distance. The diffusion model highlights that the mantle-to-rim profiles only slightly re-equilibrated through diffusion. For these profiles, the initial conditions can be directly inferred from the plagioclase An content. **d** Theoretical Sr- and Ba-in-plagioclase diffusion model for a core-to-mantle profile plotted as plagioclase An content vs. trace elements. Note the larger extent of diffusion re-equilibration undergone by these profiles. The initial conditions for the inner part of these profiles are determined using the correlation between the plagioclase An content and the trace elements from Grocolas and Müntener (2024)

Three plagioclase zones have been recognised in the Western Adamello leucotonalite. The first zone corresponds to bright, subhedral to euhedral cores (< 1–10 vol.%) presenting resorption surfaces, patchy zones, and sometimes having their inner core completely dissolved (Fig. 2a). They have An contents ranging from An_67_ to An_87_, similar to plagioclases originating from deeper levels of the crust (e.g. Ulmer et al. 1983), while their Sr and Ba contents are relatively high (600–800 μg/g) and low (< 60 μg/g), respectively (Fig. 5). The second zone is volumetrically dominant (> 80 vol.%) and corresponds to relatively dark, subhedral to euhedral mantle (Fig. 2a, b). No zoning is observed within single grains, but they span a wide range of An contents from An_39_ to An_62_. With decreasing An, plagioclase Sr and Ba contents decrease from 880 to 500 μg/g, and from ~ 100 to ~ 50 μg/g, respectively (Fig. 5). Finally, the last zone is represented by thin (10–80 μm), discontinuous rims with low BSE intensity which accounts for ~ 10–15 vol.% of the total plagioclase volume (Fig. 2a, b). This zone exhibits a sharp decrease of the Sr (~ 480–300 μg/g) and Ba (~ 60–8 μg/g) contents with decreasing An (An_32-36_) (Fig. 5) and probably represents the last crystallisation product of the Western Adamello tonalite (Grocolas and Müntener 2024). Compositional profiles measured with LA-ICP-MS and NanoSIMS, which are the same within uncertainty (see Electronic Supplementary Material ESM 1 for a comparison between LA-ICP-MS and NanoSIMS), across the core–mantle transition reveal a negative correlation of Sr and Ba with the An content, and vertical trends at the minimum and maximum An content values (Fig. 5a, b). This is typical of diffused profiles in plagioclase that almost reached complete re-equilibration (Fig. 5d; Dohmen et al. 2017). On the other hand, profiles measured across the mantle–rim transition exhibit a horizontal transition from ~ An_50_ to ~ An_35_, and vertical trends at the extreme An values (Fig. 5a, b). This type of relation between the plagioclase An content and trace elements is also caused by diffusion re-equilibration, albeit for shorter times than profiles from the core-mantle transition (Fig. 5c; Dohmen et al. 2017). Interestingly, spot analysis of plagioclase from the Western Adamello, done in large homogeneous zones, shows a slightly different trend with higher Sr and Ba contents at high An contents, and a more continuous negative correlation at lower An contents (Grocolas and Müntener 2024). This either reflects reduced or complete absence of diffusion in this dataset. Therefore, we use the correlation between the plagioclase An content and the trace elements from the data of Grocolas and Müntener (2024) to infer the initial conditions for diffusion modelling of the core-mantle profiles.

Plagioclase from the Laione granodiorite exhibits very similar textures and compositions as plagioclase from the Western Adamello leucotonalite. The three zones are also observed, except that (1) the An-rich (An_64-89_) cores are fewer than in the Western Adamello, (2) the volumetrically dominant mantles are heterogeneous and systematically present an oscillatory zoning with the An content ranging from An_39_ to An_65_ indicating multiple events of magma recharge, and (3) plagioclase rims are thinner (< 30 μm) and can reach lower An contents (An_21-40_), which could be related to different crystallisation pressure, melt H_2_O content, or fractional crystallisation (e.g. Yoder et al. 1957). In addition, similar relationships between the plagioclase An content and trace elements are observed, where most of the core-mantle profiles completely re-equilibrated, while the mantle-rim profiles only underwent partial re-equilibration. Similar to the Western Adamello, spot analysis data of plagioclase homogeneous zones from the Listino ring complex reveal a horizontal trend at high An contents (Electronic Supplementary Material ESM 1) indicating that diffusion did not operate at the same level as in crystal zones located close to sharp compositional changes. As such, the correlation between the plagioclase An content and trace elements observed from spot analysis is used to infer the initial conditions of the diffusion modelling.

## Thermometry

**Fig. 6:**
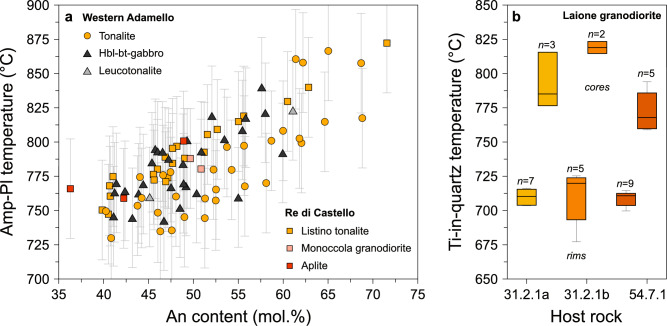
Thermometry used to infer the initial temperature of the diffusion modelling. **a** Amphibole-plagioclase temperatures (°C) vs. plagioclase An content (mol.%) for various lithologies from the Western Adamello and southern Re di Castello. The amphibole-plagioclase temperatures were calculated using the edenite-richterite thermometer of Holland and Blundy (1994) and a pressure of 250 MPa. **b** Ti-in-quartz temperatures (°C) for the three studied Laione granodiorite samples represented as box-and-whiskers. The calculated temperatures were calculated following Wark and Watson (2006) and were distinguished as a function of the spot location. *Amp*, amphibole; *Pl*, plagioclase

In this section, we use thermometers based on mineral chemistry to assess the temperature conditions at the onset of diffusion for quartz and plagioclase. Amphibole chemistry from the Western Adamello tonalite and Listino ring complex reveals that the temperature-dependent edenite exchange controlled their chemical evolution (Grocolas and Müntener 2024; Electronic Supplementary Material ESM 1). We applied the pressure-dependent amphibole–plagioclase thermometer of Holland and Blundy (1994) to determine plagioclase crystallisation temperatures, whose typical uncertainties associated with the thermometer calibration and in situ analyses are ~ 35–40 °C. We used a pressure of 250 MPa based on previous studies investigating the metamorphic contact aureole of the Western Adamello (Floess and Baumgartner 2013) and the normative composition of granitic dikes (John and Blundy 1993). As the magma was probably undersaturated with respect to quartz upon amphibole saturation and crystallisation, we employed the edenite-richterite thermometer. The resulting equilibrium temperatures range from 724 to 865 °C for the Western Adamello, and from 746 to 885 °C for the Listino ring complex (Fig. 6a). The calculated temperatures are correlated with the plagioclase An content with a residual error of ~ 15 °C. As such, we either use (1) the temperature calculated directly from the amphibole–plagioclase touching pairs, or (2) the observed correlation between the calculated temperature and the plagioclase An content when no amphibole was in contact with plagioclase. The uncertainty on the initial temperature for diffusion modelling was kept to ± 15 °C based on the standard deviation of the calculated temperatures.

The crystallisation temperature of quartz is inferred by using the temperature dependence of Ti incorporation into the quartz crystal lattice (e.g. Huang and Audétat 2012; Thomas et al. 2010, 2015; Wark and Watson 2006; Zhang et al. 2020). To accurately determine the crystallisation temperature of quartz, the Ti activity (*a*TiO_2_) must be constrained. To do so, we model the Ti chemical potential (*μ*TiO_2_) evolution during the differentiation of the Laione granodiorite using Perple_X (Connolly 2005, 2009) coupled with recently published thermodynamic solution models (Holland et al. 2018). The calculations successfully reproduce the phase proportions observed in the Laione granodiorite, as well as the modal proportions of hydrous crystallisation experiments (Marxer and Ulmer 2019). The reader is referred to the study of Grocolas and Müntener (2024) for further discussion concerning the model benchmark. To calculate *a*TiO_2_ from *μ*TiO_2_, we use Eq. 7:

7 $$ a_{{{\text{TiO}}_{2} }} = {\text{exp}}\left( { - \frac{{G_{{{\text{TiO}}_{2} }}^{P,T} - \mu_{{{\text{TiO}}_{2} }}^{P,T} }}{RT}} \right), $$

where *P* is the pressure (Pa), *T* is the temperature (K), ${\mu }_{{\text{TiO}}_{2}}^{P,T}$ is the chemical potential of rutile (J mol^−1^), ${G}_{{\text{TiO}}_{2}}^{P,T}$ is the Gibbs free energy of rutile (J), and *R* is the universal gas constant (J mol^−1^ K^−1^). In a first step, a pressure of 250 MPa is assumed based on various barometric estimates obtained across the Adamello batholith (e.g. Floess and Baumgartner 2013; John and Blundy 1993). Perple_X modelling reveals that, at the onset of quartz crystallisation (~ 715 °C), the calculated melt *a*TiO_2_ is 0.57 and decreases to 0.52 with increasing crystallinity. The crystallisation temperatures of the quartz cores and rims are then calculated using the parameterisation of Wark and Watson (2006). Given the uncertainties on the experimental fit and the analytical conditions, the uncertainty of the calculated temperatures ranges from 11 to 14 °C. The calculated temperatures for the quartz bright cores are 797 ± 47 °C (2σ), while the darker rims yield lower temperatures (717 ± 36 °C) (Fig. 6b). These results are consistent with both Perple_X modelling and experimental data using a starting material from the Adamello batholith (Marxer and Ulmer 2019), and fall within uncertainty of the pressure-dependent calibration of Huang and Audétat (2012). In contrast, recent calibrations (e.g. Kirkpatrick et al. 2025; Osborne et al. 2022) yield significantly lower temperatures (≤ 540 °C), about 170 °C below those inferred from experiments and Perple_X modelling. These unrealistically low temperatures are likely an artefact of the high-pressure range (1.0–2.5 GPa) used for those calibrations. We therefore argue that such calibrations are not applicable for determining quartz crystallisation temperatures in low-pressure granitic plutons. Accordingly, we adopt a starting temperature of 715 ± 15 °C for subsequent diffusion modelling.

Unlike the temperatures obtained from quartz rims, those calculated from core compositions are ~ 80 °C higher compared to Perple_X modelling and experimental data. The quartz stability field expands at higher pressure and lower *f*H_2_O. However, initial quartz crystallisation at higher pressures seems unlikely, as quartz typically saturates at temperatures below the rheological lock-up, at which stage efficient magma transport from higher pressures is hindered. One possibility is that these high-temperature quartz cores may have formed at the onset of the biotite-forming peritectic reaction, when *f*H_2_O is still relatively low. Alternatively, the Laione granodiorite could represent remobilised crystal mushes from the Listino ring complex, reheated during emplacement of the tonalite of Malga Listino. This thermal event could have lowered *f*H_2_O, resulting in quartz saturation at higher temperature.

## Diffusion calculations

In this section, we model diffusion in quartz and plagioclase to extract cooling rates and crystal–melt segregation timescales. This is done by using (1) the initial concentrations inferred from the measured, diffused profiles for quartz, and the correlation between the An content and trace elements observed from spot analysis for plagioclase, and (2) the initial temperatures calculated using the Ti concentration in quartz and the equilibrium between amphibole and plagioclase touching pairs.

### Cooling rates

#### Laione granodiorite

Quartz, alkali feldspar and low-An plagioclase represent the final crystallising phases of metaluminous and peraluminous intermediate to felsic melts (e.g. Johnson and Rutherford 1989). Textural relationships and phase proportions suggest that quartz Ti-poor rims from the Laione granodiorite probably crystallised at a melt fraction between 15 and 30 vol.%, before the interstitial melt reached the granite minimum. This indicates that, after crystallisation of the quartz rims, the Laione granodiorite did not experience prolonged times at suprasolidus temperatures. As such, we model the cooling rate of the Laione granodiorite by modelling the compositional profiles of Ti in quartz using the diffusion coefficients of Cherniak et al. (2007), Jollands et al. (2020) and Audétat et al. (2021, 2023). An exponential temperature decrease was imposed, and different cooling rates were tested by varying a factor within the exponential term.

**Fig. 7:**
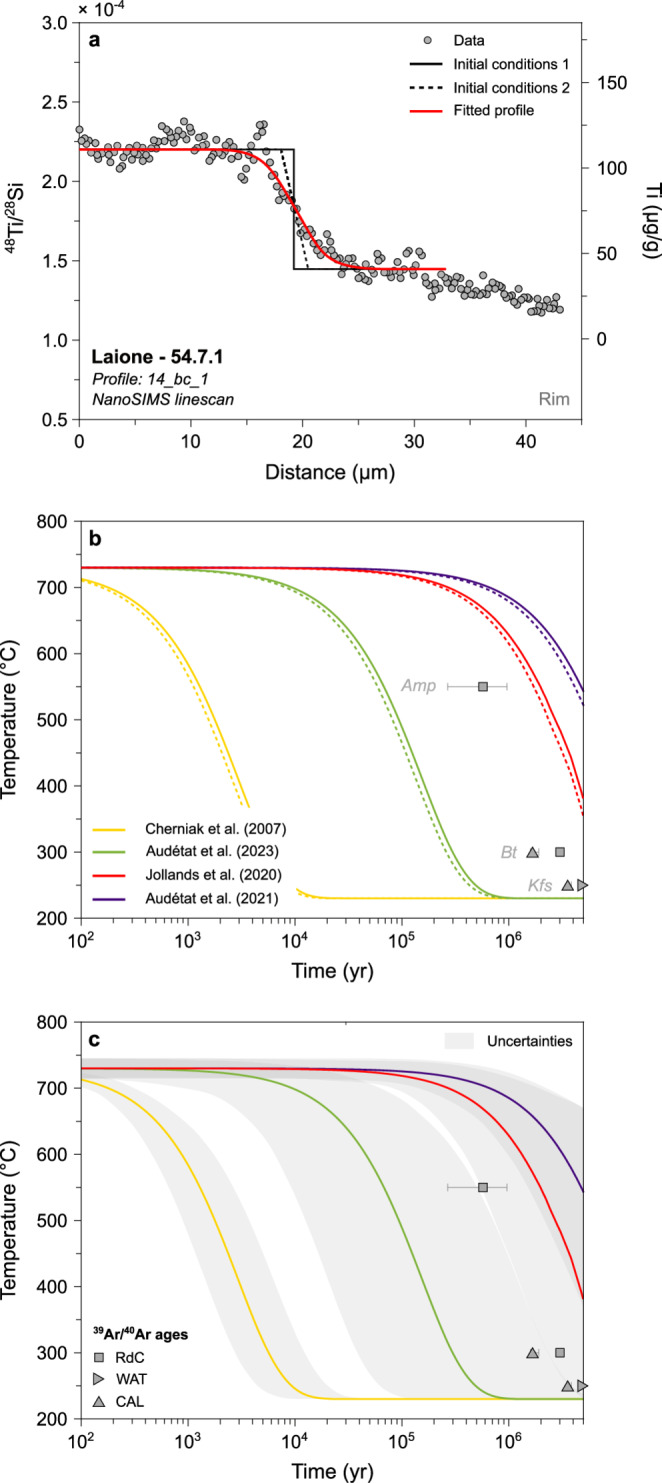
Results of Ti-in-quartz diffusion modelling used to infer cooling rates. **a** ^48^Ti/^28^Si and Ti (μg/g) vs. distance (μm). The black dotted line and continuous line represent the two initial conditions tested in the model, and the red line corresponds to the best fit to the data (grey). **b** Temperature (°C) evolution through time (yr) in logarithmic scale, with the best fits using the two different initial conditions represented. The ^39^Ar/^40^Ar ages obtained on minerals with different closure temperatures (Schaltegger et al. 2019) are represented for comparison. **c** Same figure as **b**, but with the grey zones representing the uncertainty envelopes around the best-fit cooling paths inferred from the step-like initial profile modelled using a Monte Carlo scheme

**Fig. 8:**
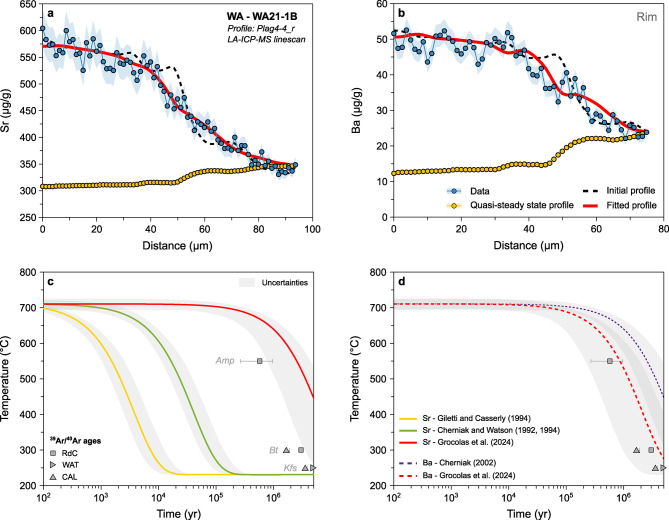
Results of Sr- and Ba-in-plagioclase diffusion modelling used to infer cooling rates. **a** Sr (μg/g) vs. distance (μm). The black dotted line represents the initial conditions, the red line corresponds to the best fit to the data (blue), and the yellow data points are for the calculated quasi-steady state profile. **b** Ba (μg/g) vs. distance (μm). **c** Temperature (°C) evolution through time (yr) in logarithmic scale, with the best fits using different diffusion coefficients for Sr in plagioclase (Cherniak and Watson 1992, 1994; Giletti and Casserly 1994; Grocolas et al. 2025) represented. The grey zones represent the uncertainty envelopes modelled using a Monte Carlo scheme. The ^39^Ar/^40^Ar ages obtained on minerals with different closure temperatures (Schaltegger et al. 2019) are represented for comparison. **d** Temperature (°C) evolution through time (yr) in logarithmic scale, with the best fits using different diffusion coefficients for Ba in plagioclase (Cherniak 2002; Grocolas et al. 2025) represented. The grey zones represent the uncertainty envelopes modelled using a Monte Carlo scheme. The ^39^Ar/^40^Ar ages obtained on minerals with different closure temperatures (Schaltegger et al. 2019) are represented for comparison

**Fig. 9:**
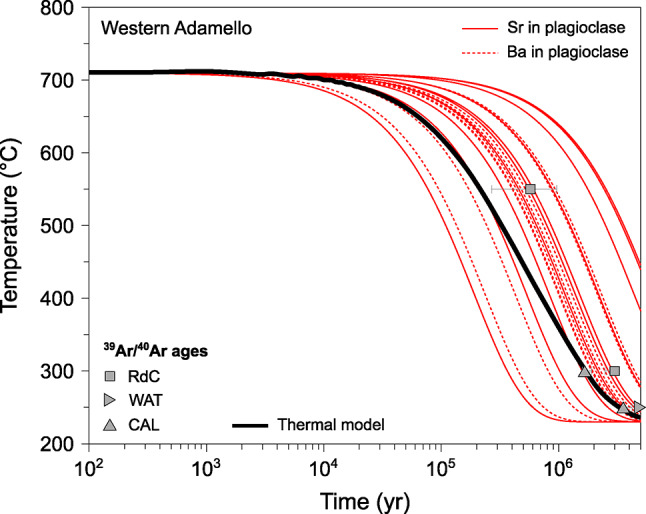
Compilation of the cooling rates obtained from Sr- and Ba-in-plagioclase diffusion modelling on 10 plagioclase crystals using the diffusivities of Grocolas et al. (2025) from Western Adamello leucotonalites compared to cooling rates inferred from mineral ^39^Ar/^40^Ar ages (Schaltegger et al. 2019) and thermal modelling

The two different initial conditions used to model diffusion in quartz (Fig. 7a) yield very similar cooling rates with a temperature difference of < 20 °C after 1 Myr of diffusion (Figs. 8 and 9). As such, only the initial conditions using a step-function profile will be further used and discussed for clarity. The cooling rates obtained from diffusion of Ti in quartz span ~ 3.5 orders of magnitude as a function of the diffusion coefficients used for modelling (Fig. 7). No clear difference is observed between the different samples. The time required for quartz crystals to cool by 100 °C from the initial conditions is ~ 0.5–2.5 kyr using the diffusion coefficients of Cherniak et al. (2007), ~ 26–130 kyr using Audétat et al. (2023), ~ 0.8–4.1 Myr using Jollands et al. (2020), and ~ 1.8–9.0 Myr using Audétat et al. (2021) (Fig. 7; Electronic Supplementary Material ESM 1). The cooling rates obtained using the diffusion coefficients of Audétat et al. (2021, 2023) and Jollands et al. (2020) are within one order of magnitude of the cooling rates inferred from ^39^Ar/^40^Ar mineral ages (Fig. 7b-d). On the other hand, the diffusivities of Cherniak et al. (2007) yield cooling rates > 2 orders of magnitude faster than the ^39^Ar/^40^Ar data. It is important to note that the temperatures associated with the ^39^Ar/^40^Ar ages correspond to closure temperatures, which depend on several parameters, including Ar diffusivity, mineral geometry, and the cooling rate itself (e.g. Dodson 1973; Schaen et al. 2020). Although estimating uncertainties is challenging, a conservative approximation for temperature uncertainty is on the order of ~ 10–15% (Schaen et al. 2020). Such fast cooling rates obtained with the diffusivities of Cherniak et al. (2007) are unrealistic for kilometre-scale magmatic systems which usually have cooling rates ranging from ~ 50 to ~ 200 °C Myr^−1^ (e.g. Annen et al. 2006; Floess and Baumgartner 2015; Long et al. 2005; Spear and Parrish 1996). As such, we do not further consider the results obtained from the diffusion models using data from Cherniak et al. (2007).

#### Western Adamello

Low-An (~ An_30_) plagioclase from the Western Adamello crystallised in equilibrium with quartz and alkali feldspar and is part of the final crystal assemblage of the tonalitic melt (Grocolas and Müntener 2024). We model the observed profiles of Sr and Ba in plagioclase and retrieve cooling rates associated with each profile. We employ the diffusion coefficients of Giletti and Casserly (1994), Cherniak and Watson (1992, 1994) and Grocolas et al. (2025) for diffusion of Sr, and Cherniak (2002) and Grocolas et al. (2025) for diffusion of Ba in plagioclase. As mentioned above, the measured mantle-rim profiles are far from quasi-steady state and, therefore, cooling rates can be calculated.

Cooling rates obtained for the Western Adamello using Sr- and Ba-in-plagioclase diffusion depend on the employed diffusion coefficients (Fig. 8). The time required for plagioclase crystals to cool from 700 to 600 °C is ~ 27–870 yr (range corresponding to minimum and maximum values) using the Sr diffusivities of Giletti and Casserly (1994), ~ 0.3–9.1 kyr using the Sr diffusivities of Cherniak and Watson (1992, 1994), and ~ 0.04–1.4 Myr using the Sr and Ba diffusivities of Grocolas et al. (2025) and the Ba diffusivities of Cherniak (2002). The slowest cooling rates obtained from Sr and Ba diffusion modelling in plagioclase using the diffusivities of Grocolas et al. (2025) and Cherniak (2002) overlap with the cooling rates of the Western Adamello tonalite inferred from ^39^Ar/^40^Ar mineral ages (Fig. 8b-d). The cooling rates calculated using the Sr diffusion coefficients of Giletti and Casserly (1994) and Cherniak and Watson (1992, 1994) are unrealistically fast for large granitoid bodies. Differences in experimentally retrieved diffusivities are discussed in detail by Grocolas et al. (2025) and are related to surface degradation and mineral assemblage in the source powders synthesised in previous studies. As such, we only consider the results of diffusion modelling using the plagioclase diffusivities of Grocolas et al. (2025) and Cherniak (2002).

To evaluate the robustness of the inferred cooling rates, a numerical model reproducing the thermal evolution of the Western Adamello tonalite was developed. Based on the results of Floess and Baumgartner (2015), the magma reservoir was constructed as the stacking of vertical, 20-m-thick dikes during a 140-year period of magmatic activity, followed by a repose time of 2200 yr, which corresponds to a magma flux of ~ 2.5 × 10^–4^ km^3^ yr^−1^. The temperature evolution was calculated by numerically solving the heat equation in two dimensions using the explicit finite-difference method, and is representative of a point located 2 km away from the southwestern contact (Electronic Supplementary Material 1). No attempt to incorporate a hydrothermal system has been made based on the absence of hydrothermal alteration in the Western Adamello tonalite. The thermal model overlaps with the cooling rates calculated using plagioclase diffusion (Fig. 9), indicating that diffusion of Sr and Ba in plagioclase mantle-rim operated continuously and recorded the cooling of the pluton.

### Discussion

The retrieved cooling rates for the Laione granodiorite, both from the Listino ring and Blumone complexes, are similar to those from the Western Adamello using the diffusivities of Audétat et al. (2023), and slower using the diffusivities of Jollands et al. (2020) (Figs. 7 and 8; Electronic Supplementary Material ESM 1). Cooling rate is primarily a function of the average magma flux (e.g. Glazner et al. 2004; de Silva and Gosnold 2007; de Saint-Blanquat et al. 2011) and, therefore, can vary within a batholith if the magma flux changes over time. The Western Adamello covers an area of ~ 105 km^2^ and, by assuming that the ~ 3 km of vertical relief represents the reservoir thickness, a volume of ~ 315 km^3^. Floess (2013) demonstrated via high-precision U–Pb zircon dating that the Western Adamello tonalite emplaced incrementally over a time period of ~ 1.2 Myr. The resulting average magma flux is ~ 3 × 10^–4^ km^3^ yr^−1^ for the Western Adamello. Similar calculations can be done for the Listino ring (Verberne 2013) and Blumone (Schoene et al. 2012) complexes, and both resulted in an average magma flux of ~ 1.5 × 10^–4^ km^3^ yr^−1^. Given the uncertainties, these fluxes can be considered similar, which is at odds with the slow cooling rates obtained using the Ti-in-quartz diffusivities of Jollands et al. (2020) and Audétat et al. (2021). However, the different emplacement mechanisms of the Western Adamello and southern Re di Castello units likely lead to different cooling rates for similar magma fluxes. The Western Adamello tonalite was emplaced incrementally from South to North by repeated intrusions of ~ 10-m-thick vertical dikes over a time period of ~ 1.2 Myr (Floess 2013; Floess and Baumgartner 2015). On the other hand, the magmatic foliation parallel to the circular structure of the Listino ring and Blumone complexes and the progressive younging towards the centre of the structure are commonly attributed to a ballooning emplacement mechanism (John and Blundy 1993; Schoene et al. 2012; Verberne 2013). These contrasting emplacement symmetries have different cooling regimes. Indeed, Caricchi et al. (2014b) modelled the thermal evolution of magma reservoirs with ballooning and vertical emplacement mechanisms and, by comparing the thermal structures after 100 kyr, they concluded that (1) a concentric growth like the southern Re di Castello leads to an isotropic heat redistribution, whereas (2) plutons emplaced by stacking of vertical dikes like the Western Adamello tonalite exhibit an ellipsoidal thermal structure with faster cooling rates in the horizontal direction. As such, a potential difference in calculated cooling rates between the Western Adamello and the Listino ring and Blumone complexes could be related to the different emplacement mechanisms. Therefore, although the Ti-in-quartz diffusivities from Audétat et al. (2023) yield similar cooling rates for the southern Re di Castello as the Western Adamello tonalite, the slower cooling rates obtained using the diffusivities of Jollands et al. (2020) and Audétat et al. (2021) cannot be excluded a priori.

### Crystal–melt segregation timescales

**Fig. 10:**
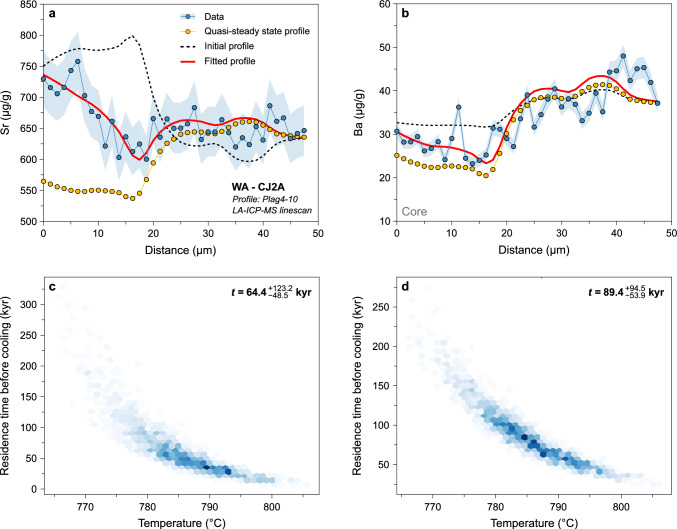
Results of Sr- and Ba-in-plagioclase diffusion modelling used to infer crystal residence times prior to crystal–melt segregation. **a** Sr (μg/g) vs. distance (μm). The black dotted line represents the initial conditions, the red line corresponds to the best fit to the data (blue), and the yellow data points are for the calculated quasi-steady state profile. **b** Ba (μg/g) vs. distance (μm). **c**, **d** Results of the Monte Carlo simulation for Sr and Ba diffusion, respectively, represented as temperature (°C) vs. time (kyr). See main text for explanations regarding diffusion modelling and Monte Carlo resampling

Xenocrysts and antecrysts are commonly found in plutonic and volcanic rocks and are indicative of open, dynamic systems. The plagioclase cores having high-An (An_70-90_) compositions having resorption surfaces found in the Western Adamello leucotonalite have been previously interpreted as antecrysts sampled in deeper parts of the crust during magma ascent (Grocolas and Müntener 2024). Similar observations and conclusions can be made for the high-An plagioclase cores occurring in the Laione granodiorite. The diffusive re-equilibration between the plagioclase cores (~ An_70-90_) and mantles (~ An_40-65_) can therefore be used to infer the plagioclase mantle residence time within the tonalitic and granodioritic mushes before crystallisation of the plagioclase rim (Fig. 10).

**Fig. 11:**
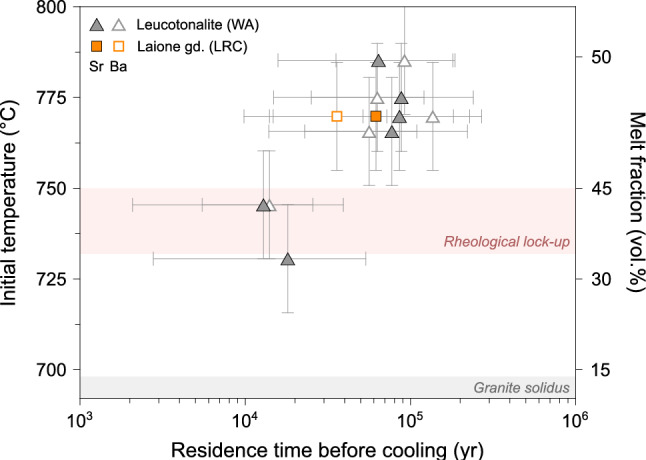
Compilation of the crystal–melt segregation timescales obtained by diffusion modelling of Sr and Ba in plagioclase using the diffusivities of Grocolas et al. (2025) from the Western Adamello leucotonalite and Laione granodiorite represented as initial temperature (°C) and melt fraction (vol.%) vs. residence time before cooling (yr). The rheological lock-up for a tonalitic starting material (Marxer and Ulmer 2019) and the water-saturated granite solidus at 200 MPa (Johannes and Holtz 2012) are represented for comparison

Diffusion modelling of Sr and Ba in plagioclase core to mantle yielded timescales ranging from ${13}_{-11}^{+27}$ to ${132}_{-80}^{+140}$ kyr for the Western Adamello leucotonalite, and ${36}_{-21}^{+37}$ to ${63}_{-53}^{+121}$ kyr for the Laione granodiorite using the diffusion coefficients of Grocolas et al. (2025) (Fig. 11). In detail, modelling of Sr and Ba in plagioclase yield the same timescales within uncertainty, with a difference ranging from 2 to 53%. Interestingly, the lowest initial temperatures coincide with the rheological lock-up (< 45 vol.% melt) temperature (Marxer and Ulmer 2019) and correspond to the shortest calculated timescales (< 20 kyr; Fig. 11). The other calculated times between plagioclase mantle crystallisation and the solidus range from ~ 40 to ~ 110 kyr, with no other distinction observed between those two groups. Within this time interval, the calculated cooling rates vary from ~ 300 to ~ 1,000 °C Myr^−1^ based on the minimum and maximum values of initial temperature and residence time. The calculated residence times above the rheological lock-up (~ 10^4^–10^5^ yr) are comparable to the thermal model developed in this study (Figs. 9 and 11) and those from previous studies (e.g. Annen et al. 2006; Caricchi et al. 2014a). These models highlight that the long-term (> 10^5^ yr) thermal evolution of magma reservoirs is a first-order function of the time-averaged rate of magma input. For relatively high magma fluxes (~ 10^–2^ km^3^ yr^−1^), thermal models predict that eruptible magma (i.e. above the rheological lock-up) continuously accumulates within the magma reservoir, whereas for lower magma input such as in the Adamello batholith (~ 10^–4^ km^3^ yr^−1^), eruptible magma is only sporadically present for time periods of ≤ 10^5^ yr (e.g. Annen 2009). In addition, Karakas et al. (2017) showed that in transcrustal magmatic systems, the development of extensive lower crustal mush zones modify the thermal budget of the upper crust. The magma fluxes required to sustain shallow magma reservoirs is therefore reduced, allowing systems like the Adamello batholith to store mobile magma for tens of thousands of years.

## Comparison with volcanic timescales

In this section, we compare diffusion timescales obtained in (1) well-studied volcanic rocks using different diffusivities, (2) with magma chamber lifespans as determined by high-precision zircon U–Pb dating, and (3) with the crystal–melt segregation timescales obtained in the Adamello batholith.

### Which diffusion coefficients?

**Fig. 12:**
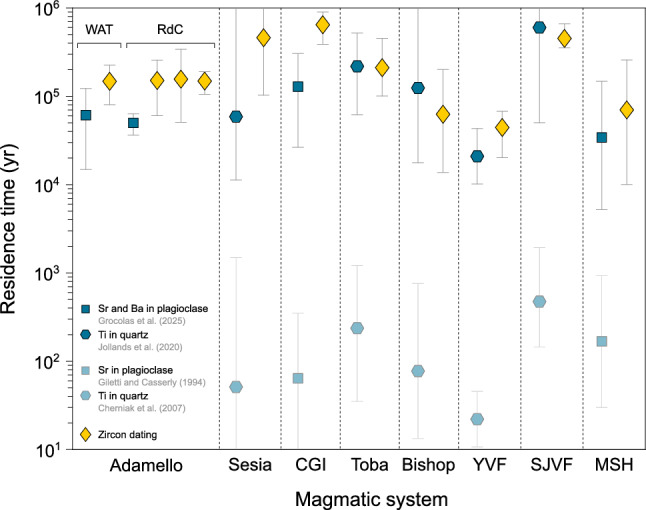
Compilation of crystal residence time prior to eruption (or cooling) obtained for different silicic volcanoes worldwide through diffusion modelling, or high-precision U–Pb zircon dating, and comparison with the timescales obtained in this study. Symbols represent different minerals, colours represent the technique employed to infer timescales, and the symbol is positioned at the average value. Ti-in-quartz and Sr-in-plagioclase timescales were recalculated using the diffusion coefficients of Jollands et al. (2020) and Grocolas et al. (2025), respectively. Residence times inferred from diffusion in quartz and plagioclase generally span 10^4^ to 10.^6^ yr, similar to the zircon crystallisation timespan. Magmatic systems used in this compilation include: Adamello batholith (Broderick et al. 2015; Floess 2013; Schoene et al. 2012; Verberne 2013; this study); Sesia magmatic system (Karakas et al. 2019; Tavazzani et al. 2020, 2023); Cerro Galán ignimbrite (CGI) (Folkes et al. 2011; Lubbers et al. 2022); Younger Toba Tuff (Reid and Vazquez 2017; Szymanowski et al. 2023; Wang et al. 2023); Bishop Tuff (Chamberlain et al. 2014a,b; Gualda et al. 2012); Yellowstone Volcanic Field (YVF) (Shamloo and Till 2019; Wotzlaw et al. 2014); San Juan Volcanic Field (SJVF) (Brückel et al. 2023; Wotzlaw et al. 2013); Mount St. Helens (MSH) (Claiborne et al. 2010; Schlieder et al. 2022)

Recent advances show that Ti diffusion rates in quartz might be slower by ~ 2–3 orders of magnitude (Audétat et al. 2021, 2023; Jollands et al. 2020) than previously determined (Cherniak et al. 2007). Measured Ti-in-quartz profiles usually display complex reverse zoning and are located in the mantle part of millimetre-sized quartz phenocrysts (e.g. Brückel et al. 2023; Shamloo and Till 2019; Tavazzani et al. 2020; Wang et al. 2023), except for the Bishop Tuff where the profiles measured by Chamberlain et al. (2014a) are located in the outermost ~ 100–200 μm. Although rapid growth may play a role (Pamukcu et al. 2016) in generating such reverse zoning, it is typically interpreted as reflecting the magma recharge event preceding volcanic eruption (e.g. Matthews et al. 2012; Wark et al. 2007). The timescales inferred from these studies range from ~ 10^2^ yr using the diffusivities of Cherniak et al. (2007), to ~ 10^5^ yr employing diffusion coefficients from Jollands et al. (2020) and Audétat et al. (2021, 2023) (Fig. 12). The faster diffusion timescales are similar to those from some mafic systems (e.g. Costa et al. 2020). However, magmatic processes in felsic systems are generally slowed by the highly viscous character of silicic magmas. Furthermore, the large Ti–rich rim zones of volcanic quartz crystals often display an internal oscillatory zoning (e.g. Shamloo and Till 2019). This feature is typical of multiple recharges of magma having less differentiated compositions (e.g. Matthews et al. 2012; Wark et al. 2007) and reflects the protracted magmatic history of those crystals, which favours long residence times prior to eruption. This is further supported by thermal models (e.g. Bachmann and Bergantz 2004) and zircon thermochronology (e.g. Barboni et al. 2016) studies emphasising that silicic magma reservoirs are stored for tens to hundreds of thousands of years below ~ 750 °C.

A similar reassessment has been conducted for Sr and Ba in plagioclase, with Grocolas et al. (2025) demonstrating that Sr diffusion rates in plagioclase were ~ 2–3 orders of magnitude slower than previously determined by Giletti and Casserly (1994) and Cherniak and Watson (1992, 1994), while Ba diffusion rates were similar to those defined by Cherniak (2002). As discussed above, the Sr diffusivities from previous studies yield unreasonably fast cooling rates for the Adamello batholith, 1.5 to 2.5 orders of magnitude faster than the cooling rates obtained from ^39^Ar/^40^Ar mineral ages. Furthermore, Grocolas et al. (2025) recalculated diffusion timescales with the new Sr and Ba diffusivities for the Cerro Galán and Santorini volcanic systems and obtained similar, long (> 10^4^ yr) times using both elements. The same long timescales within uncertainty were obtained using the Ba-in-plagioclase diffusivities of Cherniak (2002). For these reasons and based on experimental considerations discussed in previous studies (Grocolas et al. 2025; Jollands et al. 2020), only the diffusion timescales using the Sr- and Ba-in-plagioclase diffusivities of Grocolas et al. (2025), and the Ti-in-quartz diffusivities of Jollands et al. (2020) will be used for further discussion.

### Similarity with zircon timescales

High-precision chemical abrasion–isotopic dilution–thermal ionisation mass spectrometry (CA-ID-TIMS) dating is typically associated with uncertainties of ~ 20 kyr and therefore represents the ideal analytical technique to resolve magmatic processes operating over timescales of 10^4^–10^6^ yr (e.g. Schaltegger et al. 2009). Moreover, dating of young (< 1 Ma) zircons using conventional in situ techniques (e.g., SIMS) also yields ages with reasonable uncertainties (< 50 kyr; e.g. Chamberlain et al. 2014b). We compiled high-precision U–Pb zircon ages for the Adamello batholith and well-studied caldera-forming eruptions and compare it with the crystal residence times inferred from diffusion modelling (Fig. 12). The crystal residence time before cooling in the Adamello batholith, interpreted as crystal–melt segregation timescales, ranges from ~ 10^4^ to ~ 10^5^ yr, while zircon records crystallisation timescales of ~ 10^5^ yr (Broderick et al. 2015; Floess 2013; Schaltegger et al. 2009; Schoene et al. 2012; Verberne 2013). Similar conclusions can be drawn from large-volume, caldera-forming rhyolitic eruptions, where diffusion timescales are usually similar to zircon crystallisation timespan.

However, the zircon age distribution produced in magma reservoir and measured via CA-ID-TIMS is a function of the zircon saturation temperature and the reservoir cooling rate to the solidus, potentially leading to different interpretations between magmatic systems. If zircon saturation is reached at the emplacement depth, these grains are called “autocrysts” and their age distribution documents the magma reservoir lifespan. On the other hand, magmas emplaced at temperatures below the zircon saturation temperature may carry zircon crystals from lower parts of the crust genetically related (“antecrysts”) or unrelated (“xenocrysts”) to the parental magmas (Miller et al. 2007). This second endmember typically results in a zircon crystallisation history longer than the upper crustal magma body. In the Adamello batholith, a zircon saturation temperature of ~ 800 °C has been experimentally determined for a tonalite with ~ 61 wt.% SiO_2_ (Marxer and Ulmer 2019), which is consistent with the highest calculated Ti-in-zircon temperatures ranging from 790 to 810 °C in the Western Adamello (Electronic Supplementary Material ESM 2). This is below the emplacement temperature of ~ 900 °C for the Western Adamello tonalite (Floess and Baumgartner 2015; Grocolas and Müntener 2024). Hence, zircons from the Western Adamello are autocrysts and their crystallisation ages most likely represent the duration between zircon saturation (~ 800 °C) and the H_2_O-saturated granite solidus (~ 680 °C).

Constraining the emplacement mechanism and temperature of parental magmas associated with volcanic eruptions is challenged by the rare exposure of their plutonic roots. As such, a rigorous comparison of zircon saturation temperatures with emplacement temperatures remains elusive, which prevents determination of the timing of zircon saturation (e.g. Ratschbacher et al. 2024). The zircon age spreads obtained via high-precision CA-ID-TIMS dating are therefore considered as maximum residence times since there is no robust method to differentiate antecrysts from autocrysts. These maximum zircon residence times before eruption are similar within uncertainty to the diffusion timescales, but some exhibit higher values than those derived from diffusion modelling. This small difference may arise from the higher zircon saturation temperatures compared to the initial temperatures used in diffusion modelling, resulting in the presence of antecrystic zircons mechanically expanding the zircon apparent residence time. Regardless, these similar timescales demonstrate the ability of plagioclase and quartz to record residence times prior to melt segregation and extraction. In addition, previous studies showed that diffusion in outermost crystal rims can also record mixing-to-eruption timescales (e.g. Till et al. 2015), highlighting the importance of detailed petrographic observations prior to diffusion modelling.

### Volcanic and plutonic timescales

The crystal–melt segregation timescales obtained through diffusion modelling in this study are, to our knowledge, the first determined in plutonic rocks. Interestingly, these plutonic timescales (10^4^–10^5^ yr) are comprised within the same range as the crystal residence times prior to volcanic eruption (10^4^–10^6^ yr) (Fig. 12). Such similarity raises questions regarding the nature of the trigger of volcanic eruptions, as opposed to the scarcity of volcanic products originating from the Adamello batholith (Lu et al. 2018). Most studies suggest, based on thermal modelling, that the eruptability of a given magmatic system is largely controlled by the average magma flux (Caricchi et al. 2014a; Degruyter and Huber 2014). This control is well demonstrated by the contrasting magma fluxes inferred from the Adamello batholith and plutonic bodies in general (~ 10^–4^ km^3^ yr^−1^), and the ones associated with caldera-forming eruptions which are typically ~ 2 orders of magnitude higher (Costa 2008). In addition, the phenocrysts included in these volcanic rocks often present reverse zoning at the crystal rims, revealing the important role of mafic rejuvenation prior to eruption (Bachmann et al. 2002; Murphy et al. 2000; Vazquez and Reid 2004; Wark et al. 2007). In this thermal rejuvenation scenario, crystal–rich mushes are re-heated and subsequently undergo convection and homogenisation (e.g. Couch et al. 2001). On the other hand, the scarcity of re-heating event in plutonic systems, as evidenced by the scarcity of reverse zoning in those rocks, may also hinder large volumes of melt to be segregated and erupted. We propose that 10^4^–10^6^ yr represents the ideal time to assemble a thermally stable magma reservoir able to internally segregate interstitial melt and extract it to shallower depths. Our calculated timescales suggest that this thermal maturation time is largely independent of the average magma flux, which is supported by thermal modelling (e.g. Karakas et al. 2017) whereby the development of extensive lower crustal mush zones controls the thermal budget of the upper crust.

## Conclusions

The compositional zoning of plagioclase and quartz from the Western Adamello leucotonalite and Laione granodiorite record the thermal evolution of the Adamello batholith and was used to infer residence times and cooling rates. Microscopic observations coupled with mineral chemistry suggest that plagioclase from both units are composed of high-An, inherited cores, volumetrically dominant mantles with intermediate An values, and thin, low-An rims. In addition to the plagioclase mantle-to-rim profiles, the Ti zoning present in quartz is used to infer the cooling rates of the different units. The initial temperatures used to model diffusion were constrained using the compositions of amphibole–plagioclase pairs and the Ti concentration of quartz rims and yielded temperatures of 700–730 °C. The modelled nonlinear cooling rates are relatively slow with a cooling from 700 to 600 °C attained in ~ 400 kyr in the Western Adamello, and > 1 Myr for the Laione granodiorite, in reasonable agreement with thermal models and ^39^Ar/^40^Ar mineral ages determined in previous studies. Furthermore, the plagioclase core-to-mantle profiles were reproduced by using the calculated cooling rates and amphibole–plagioclase equilibrium temperatures of 730–780 °C. The extracted timescales are in the range of ~ 20–110 kyr, which corresponds to the time period between the crystallisation of the plagioclase mantle and the rim. These crystal residence times above the solidus are similar to (1) crystal residence times before caldera-forming eruptions and (2) zircon crystallisation timespan for the same plutonic and volcanic systems. Consequently, such timescales (10^4^–10^6^ yr) probably represent the ideal time to form a thermally stable magma reservoir able to segregate interstitial melt, regardless of the average magma fluxes. The latter instead controls the total amount of segregated melt and, thus, the eruptability of a magma reservoir (e.g. Caricchi et al. 2014a).

## Supplementary Information

Below is the link to the electronic supplementary material.

Supplementary file1 (PDF 4836 KB)

Supplementary file2 (XLSX 476 KB)

## Data Availability

Datasets for this research are included in the Electronic Supplementary Material.

## References

[CR1] Annen C (2009) From plutons to magma chambers: thermal constraints on the accumulation of eruptible silicic magma in the upper crust. Earth Planet Sci Lett 284:409–416. 10.1016/j.epsl.2009.05.006

[CR2] Annen C, Blundy JD, Sparks RSJ (2006) The genesis of intermediate and silicic magmas in deep crustal hot zones. J Petrol 47:505–539. 10.1093/petrology/egi084

[CR3] Armstrong JT (1995) CITZAF–A package of correction programs for the quantitative electron microbeam X-ray analysis of thick polish materials, thin-films, and particles. Microbeam Anal 4:177–200

[CR4] Audétat A, Garbe-Schönberg D, Kronz A, Pettke T, Rusk B, Donovan JJ, Lowers HA (2015) Characterisation of a natural quartz crystal as a reference material for microanalytical determination of Ti, Al, Li, Fe, Mn, Ga and Ge. Geostand Geoanalytical Res 39:171–184. 10.1111/j.1751-908X.2014.00309.x

[CR5] Audétat A, Miyajima N, Wiesner D, Audinot JN (2021) Confirmation of slow Ti diffusion in quartz by diffusion couple experiments and evidence from natural samples. Geology 49:963–967. 10.1130/G48785.1

[CR6] Audétat A, Schmitt AK, Njul R, Saalfeld M, Borisova A, Lu Y (2023) New constraints on Ti diffusion in quartz and the priming of silicic volcanic eruptions. Nat Commun 14:4277. 10.1038/s41467-023-39912-5 37460528 10.1038/s41467-023-39912-5 PMC10352339

[CR7] Audétat A (2021) Comment on “Ti-in-quartz thermobarometry and TiO_2_ solubility in rhyolitic melts: new experiments and parametrization” by Zhang et al. [Earth Planet Sci Lett 538:116213]. Earth Planet Sci Lett 561:116847. 10.1016/j.epsl.2021.116847

[CR8] Bachmann O, Bergantz GW (2004) On the origin of crystal–poor rhyolites: extracted from batholitic crystal mushes. J Petrol 45:1565–1582. 10.1093/petrology/egh019

[CR9] Bachmann O, Dungan MA, Lipman PW (2002) The Fish Canyon magma body, San Juan volcanic field, Colorado: rejuvenation and eruption of an upper-crustal batholith. J Petrol 43:1469–1503. 10.1093/petrology/43.8.1469

[CR10] Barboni M, Schoene B (2014) Short eruption window revealed by absolute crystal growth rates in a granitic magma. Nat Geosci 7:524–528. 10.1038/ngeo2185

[CR11] Barboni M, Boehnke P, Schmitt AK, Harrison TM, Shane P, Bouvier AS, Baumgartner LP (2016) Warm storage for arc magmas. Proc Natl Acad Sci 113:13959–13964. 10.1073/pnas.1616129113 27799558 10.1073/pnas.1616129113 PMC5150383

[CR12] Bindeman IN, Davis AM, Drake MJ (1998) Ion microprobe study of plagioclase-basalt partition experiments at natural concentration levels of trace elements. Geochim Cosmochim Acta 62:1175–1193. 10.1016/S0016-7037(98)00047-7

[CR13] Broderick C, Wotzlaw JF, Frick DA, Gerdes A, Ulianov A, Günther D, Schaltegger U (2015) Linking the thermal evolution and emplacement history of an upper-crustal pluton to its lower-crustal roots using zircon geochronology and geochemistry (southern Adamello batholith, N. Italy). Contrib Mineral Petrol. 170:28. 10.1007/s00410-015-1184-x

[CR14] Brückel K, Lundstrom CC, Ackerson M, Campe C (2023) Testing the limits of Ti-in-Quartz thermometry and diffusion modelling to determine the thermal history of the Fish Canyon Tuff. J Petrol 64:egad082. 10.1093/petrology/egad082

[CR15] Callegari E, Brack P (2002) Geological map of the tertiary Adamello batholith (northern Italy): explanatory notes and legend. Mem Sci Geol 54:19–49

[CR16] Caricchi L, Annen C, Blundy J, Simpson G, Pinel V (2014a) Frequency and magnitude of volcanic eruptions controlled by magma injection and buoyancy. Nat Geosci 7:126–130. 10.1038/ngeo2041

[CR17] Caricchi L, Simpson G, Schaltegger U (2014b) Zircons reveal magma fluxes in the Earth’s crust. Nature 511:457–461. 10.1038/nature13532 25056063 10.1038/nature13532

[CR18] Cashman KV, Sparks RSJ, Blundy JD (2017) Vertically extensive and unstable magmatic systems: a unified view of igneous processes. Science 355:eaag3055. 10.1126/science.aag3035 28336610 10.1126/science.aag3055

[CR19] Chamberlain KJ, Morgan DJ, Wilson CJ (2014a) Timescales of mixing and mobilisation in the Bishop Tuff magma body: perspectives from diffusion chronometry. Contrib Mineral Petrol 168:1–24. 10.1007/s00410-014-1034-2

[CR20] Chamberlain KJ, Wilson CJ, Wooden JL, Charlier BL, Ireland TR (2014b) New perspectives on the Bishop Tuff from zircon textures, ages and trace elements. J Petrol 55:395–426. 10.1093/petrology/egt072

[CR21] Chambers M, Memeti V, Eddy MP, Schoene B (2020) Half a million years of magmatic history recorded in a K-feldspar megacryst of the Tuolumne Intrusive Complex, California, USA. Geology 48:400–404. 10.1130/G46873.1

[CR22] Cherniak DJ (2002) Ba diffusion in feldspar. Geochim Cosmochim Acta 66:1641–1650. 10.1016/S0016-7037(01)00866-3

[CR23] Cherniak DJ, Watson EB (1992) A study of strontium diffusion in K-feldspar, Na-K feldspar and anorthite using Rutherford Backscattering Spectroscopy. Earth Planet Sci Lett 113:411–425. 10.1016/0012-821X(92)90142-I

[CR24] Cherniak DJ, Watson EB (1994) A study of strontium diffusion in plagioclase using Rutherford backscattering spectroscopy. Geochim Cosmochim Acta 58:5179–5190. 10.1016/0016-7037(94)90303-4

[CR25] Cherniak DJ, Watson EB, Wark DA (2007) Ti diffusion in quartz. Chem Geol 236:65–74. 10.1016/j.chemgeo.2006.09.001

[CR26] Claiborne LL, Miller CF, Flanagan DM, Clynne MA, Wooden JL (2010) Zircon reveals protracted magma storage and recycling beneath Mount St. Helens Geology 38:1011–1014. 10.1130/G31285.1

[CR27] Condomines M, Gauthier PJ, Sigmarsson O (2003) Timescales of magma chamber processes and dating of young volcanic rocks. Rev Mineral Geochem 52:125–174. 10.2113/0520125

[CR28] Connolly JAD (2005) Computation of phase equilibria by linear programming: a tool for geodynamic modeling and its application to subduction zone decarbonation. Earth Planet Sci Lett 236:524–541. 10.1016/j.epsl.2005.04.033

[CR29] Connolly JAD (2009) The geodynamic equation of state: what and how. Geochem Geophys Geosyst. 10:1–19. 10.1029/2009GC002540

[CR30] Cooper KM (2015) Timescales of crustal magma reservoir processes: insights from U-series crystal ages. Geol Soc Spec Publ 422:141–174. 10.1144/SP422.7

[CR31] Cooper KM (2019) Time scales and temperatures of crystal storage in magma reservoirs: implications for magma reservoir dynamics. Philos Trans R Soc A Math Phys Eng Sci 377:20180009. 10.1098/rsta.2018.0009 10.1098/rsta.2018.0009 PMC6335477 30966941

[CR32] Cooper KM, Kent AJ (2014) Rapid remobilization of magmatic crystals kept in cold storage. Nature 506:480–483. 10.1038/nature12991 24531766 10.1038/nature12991

[CR33] Cooper KM, Reid MR (2008) Uranium-series crystal ages. Rev Mineral Geochem 69:479–544. 10.2138/rmg.2008.69.1

[CR34] Costa F (2008) Residence times of silicic magmas associated with calderas. In: Gottsmann J, Marti J (eds) Developments in volcanology, vol 10. Amsterdam. Netherlands, Elsevier, pp 1–55

[CR35] Costa F, Chakraborty S, Dohmen R (2003) Diffusion coupling between trace and major elements and a model for calculation of magma residence times using plagioclase. Geochim Cosmochim Acta 67:2189–2200. 10.1016/S0016-7037(02)01345-5

[CR36] Costa F, Shea T, Ubide T (2020) Diffusion chronometry and the timescales of magmatic processes. Nat Rev Earth Environ 1:201–214. 10.1038/s43017-020-0038-x

[CR37] Couch S, Sparks RSJ, Carroll MR (2001) Mineral disequilibrium in lavas explained by convective self-mixing in open magma chambers. Nature 411:1037–1039. 10.1038/35082540 11429601 10.1038/35082540

[CR38] Crank J (1975) The mathematics of diffusion. Clarendon Press, Oxford

[CR39] de Silva SL, Gosnold WD (2007) Episodic construction of batholiths: Insights from the spatiotemporal development of an ignimbrite flare-up. J Volcanol Geotherm Res 167:320–335. 10.1016/j.jvolgeores.2007.07.015

[CR40] de Saint BM, Horsman E, Habert G, Morgan S, Vanderhaeghe O, Law R, Tikoff B (2011) Multiscale magmatic cyclicity, duration of pluton construction, and the paradoxical relationship between tectonism and plutonism in continental arcs. Tectonophysics 500:20–33. 10.1016/j.tecto.2009.12.009

[CR41] Degruyter W, Huber C (2014) A model for eruption frequency of upper crustal silicic magma chambers. Earth Planet Sci Lett 403:117–130. 10.1016/j.epsl.2014.06.047

[CR42] Del Moro A, Pardini GC, Quercioli C, Villa IM, Callegari E (1983) Rb/Sr and K/Ar chronology of Adamello granitoids, southern Alps. Mem Soc Geol Italiana 26:285–299

[CR43] Devoir A, Bloch EM, Müntener O (2021) Residence time of igneous garnet in Si-rich magmatic systems: Insights from diffusion modeling of major and trace elements. Earth Planet Sci Lett 560:116771. 10.1016/j.epsl.2021.116771

[CR44] Dodson MH (1973) Closure temperature in cooling geochronological and petrological systems. Contrib Mineral Petrol 40:259–274. 10.1007/BF00373790

[CR45] Dohmen R, Blundy J (2014) A predictive thermodynamic model for element partitioning between plagioclase and melt as a function of pressure, temperature and composition. Am J Sci 314:1319–1372. 10.2475/09.2014.04

[CR46] Dohmen R, Faak K, Blundy JD (2017) Chronometry and speedometry of magmatic processes using chemical diffusion in olivine, plagioclase and pyroxenes. Rev Mineral Geochem 83:535–575. 10.2138/rmg.2017.83.16

[CR47] Druitt TH, Costa F, Deloule E, Dungan M, Scaillet B (2012) Decadal to monthly timescales of magma transfer and reservoir growth at a caldera volcano. Nature 482:77–80. 10.1038/nature10706 22297973 10.1038/nature10706

[CR48] Faak K, Coogan LA, Chakraborty S (2014) A new Mg-in-plagioclase geospeedometer for the determination of cooling rates of mafic rocks. Geochim Cosmochim Acta 140:691–707. 10.1016/j.gca.2014.06.005

[CR49] Fick A (1855) Ueber diffusion. Annalen der Physik 170:59–86

[CR50] Floess D, Baumgartner LP (2013) Formation of garnet clusters during polyphase metamorphism. Terra Nova 25:144–150. 10.1111/ter.12018

[CR51] Floess D, Baumgartner LP (2015) Constraining magmatic fluxes through thermal modelling of contact metamorphism. Geol Soc Spec Publ. 422:41–56. 10.1144/SP422.8

[CR52] Floess D (2013) Contact metamorphism and emplacement of the Western Adamello tonalite. PhD thesis, University of Lausanne.

[CR53] Folkes CB, de Silva SL, Schmitt AK, Cas RA (2011) A reconnaissance of U-Pb zircon ages in the Cerro Galán system, NW Argentina: prolonged magma residence, crystal recycling, and crustal assimilation. J Volcanol Geotherm Res 206:136–147. 10.1016/j.jvolgeores.2011.06.001

[CR54] Giletti BJ, Casserly JED (1994) Strontium diffusion kinetics in plagioclase feldspars. Geochim Cosmochim Acta 58:3785–3793. 10.1016/0016-7037(94)90363-8

[CR55] Glazner AF, Bartley JM, Coleman DS, Gray W, Taylor RZ (2004) Are plutons assembled over millions of years by amalgamation from small magma chambers? GSA Today. 14:4–11. 10.17615/nspy-zk53

[CR56] Götze J, Plötze M, Habermann D (2001) Origin, spectral characteristics and practical applications of the cathodoluminescence (CL) of quartz–a review. Mineral Petrol 71:225–250. 10.1007/s007100170040

[CR57] Grocolas T, Müntener O (2024) The role of peritectic biotite for the chemical and mechanical differentiation of felsic plutonic rocks (Western Adamello, Italy). J Petrol 65:egae009. 10.1093/petrology/egae009

[CR58] Grocolas T, Bloch EM, Bouvier AS, Müntener O (2025) Diffusion of Sr and Ba in plagioclase: composition and silica activity dependencies, and application to volcanic rocks. Earth Planet Sci Lett 651:119141. 10.1016/j.epsl.2024.119141

[CR59] Grove TL, Baker MB, Kinzler RJ (1984) Coupled CaAl-NaSi diffusion in plagioclase feldspar: experiments and applications to cooling rate speedometry. Geochim Cosmochim Acta 48:2113–2121. 10.1016/0016-7037(84)90391-0

[CR60] Gualda GA, Pamukçu AS, Ghiorso MS, Anderson AT Jr, Sutton SR, Rivers ML (2012) Timescales of quartz crystallization and the longevity of the Bishop giant magma body. PLoS ONE 7:e37492. 10.1371/journal.pone.0037492 22666359 10.1371/journal.pone.0037492 PMC3364253

[CR61] Higgins MD (2006) Verification of ideal semi-logarithmic, lognormal or fractal crystal size distributions from 2D datasets. J Volcanol Geotherm Res 154:8–16. 10.1016/j.jvolgeores.2005.09.015

[CR62] Holland TJB, Blundy J (1994) Non-ideal interactions in calcic amphiboles and their bearing on amphibole-plagioclase thermometry. Contrib Mineral Petrol 116:433–447. 10.1007/BF00310910

[CR63] Holland TJB, Green EC, Powell R (2018) Melting of peridotites through to granites: a simple thermodynamic model in the system KNCFMASHTOCr. J Petrol 59:881–900. 10.1093/petrology/egy048

[CR64] Huang R, Audétat A (2012) The titanium-in-quartz (TitaniQ) thermobarometer: A critical examination and re-calibration. Geochim Cosmochim Acta 84:75–89. 10.1016/j.gca.2012.01.009

[CR65] Jochum KP, Weis U, Stoll B, Kuzmin D, Yang Q, Raczek I, Jacob DE, Stracke A, Birbaum K, Frick DA, Günther D, Enzweiler J (2011) Determination of reference values for NIST SRM 610–617 glasses following ISO guidelines. Geostand Geoanalytical Res 35:397–429. 10.1111/j.1751-908X.2011.00120.x

[CR66] John BE, Blundy JD (1993) Emplacement-related deformation of granitoid magmas, southern Adamello Massif, Italy. Geol Soc Am Bull 105:1517–1541. 10.1130/0016-7606(1993)105%3c1517:ERDOGM%3e2.3.CO;2

[CR67] Johnson MC, Rutherford MJ (1989) Experimentally determined conditions in the Fish Canyon Tuff, Colorado, magma chamber. J Petrol 30:711–737. 10.1093/petrology/30.3.711

[CR68] Jollands MC, Bloch EM, Müntener O (2020) New Ti-in-quartz diffusivities reconcile natural Ti zoning with time scales and temperature of upper crustal magma reservoirs. Geology 48:654–657. 10.1130/G47238.1

[CR69] Karakas O, Degruyter W, Bachmann O, Dufek J (2017) Lifetime and size of shallow magma bodies controlled by crustal-scale magmatism. Nat Geosci 10:446–450. 10.1038/natgeo2959

[CR70] Karakas O, Wotzlaw JF, Guillong M, Ulmer P, Brack P, Economos RC, Bergantz GW, Sinigoi S, Bachmann O (2019) The pace of crustal-scale magma accretion and differentiation beneath silicic caldera volcanoes. Geology 47:719–723. 10.1130/G46020.1

[CR71] Kirkpatrick HM, Trail D, Harrison TM, Bell EA (2025) Investigating pressur effects on Ti and Zr partitioning into zircon, quartz, and rutile at crustal temperatures. Chem Geol 673:122518. 10.1016/j.chemgeo.2024.122518

[CR72] Klein BZ, Jagoutz O, Ramezani J (2021) High-precision geochronology requires that ultrafast mantle-derived magmatic fluxes built the transcrustal Bear Valley Intrusive Suite, Sierra Nevada, California, USA. Geology 49:106–110. 10.1130/G47952.1

[CR73] LaTourette T, Wasserburg GJ (1998) Mg diffusion in anorthite: implications for the formation of early solar system planetesimals. Earth Planet Sci Lett 158:91–108. 10.1016/S0012-821X(98)00048-X

[CR74] Long LE, Castellana CH, Sial AN (2005) Age, origin and cooling history of the Coronel João Sá pluton, Bahia, Brazil. J Petrol 46:255–273. 10.1093/petrology/egh070

[CR75] Lu G, Winkler W, Rahn M, von Quadt A, Willett SD (2018) Evaluating igneous sources of the Taveyannaz formation in the Central Alps by detrital zircon U-Pb age dating and geochemistry. Swiss J Geosci 111:399–416. 10.1007/s00015-018-0302-y

[CR76] Lubbers J, Kent AJ, de Silva SL (2022) Thermal budgets of magma storage constrained by diffusion chronometry: the Cerro Galán ignimbrite. J Petrol 63:egac048. 10.1093/petrology/egac048

[CR77] Marsh BD (1981) On the crystallinity, probability of occurrence, and rheology of lava and magma. Contrib Mineral Petrol 78:85–98. 10.1007/BF00371146

[CR78] Marsh BD (1988) Crystal size distribution (CSD) in rocks and the kinetics and dynamics of crystallization: I. Theory Contrib Mineral Petrol 99:277–291. 10.1007/BF00375362

[CR79] Marxer F, Ulmer P (2019) Crystallisation and zircon saturation of calc-alkaline tonalite from the Adamello batholith at upper crustal conditions: an experimental study. Contrib Mineral Petrol 174:1–29. 10.1007/s00410-019-1619-x

[CR81] Matthews NE, Huber C, Pyle DM, Smith VC (2012) Timescales of magma recharge and reactivation of large silicic systems from Ti diffusion in quartz. J Petrol 53:1385–1416. 10.1093/petrology/egs020

[CR82] Miller JS, Matzel JE, Miller CF, Burgess SD, Miller RB (2007) Zircon growth and recycling during the assembly of large, composite arc plutons. J Volcanol Geotherm Res 167:282–299. 10.1016/j.jvolgeores.2007.04.019

[CR83] Morgan D, Blake S, Rogers N, De Vivo B, Rolandi G, Davidson J (2006) Magma chamber recharge at Vesuvius in the century prior to the eruption of AD 79. Geology 34:845–848. 10.1130/G22604.1

[CR84] Murphy M, Sparks RSJ, Barclay J, Carroll MR, Brewer T (2000) Remobilization of andesite magma by intrusion of mafic magma at the Soufriere Hills Volcano, Montserrat, West Indies. J Petrol 41:21–42. 10.1093/petrology/41.1.21

[CR85] Mutch EJF, Maclennan J, Shorttle O, Rudge JF, Neave DA (2021) DFENS: diffusion chronometry using finite elements and nested sampling. Geochem Geophys Geosyst. 22:e2020GC009303. 10.1029/2020GC009303

[CR86] Mutch EJF, Maclennan J, Madden-Nadeau AL (2022) The dichotomous nature of Mg partitioning between plagioclase and melt: implications for diffusion chronometry. Geochim Cosmochim Acta 339:173–189. 10.1016/j.gca.2022.10.035

[CR87] Nielsen RL, Ustunisik G, Weinsteiger AB, Tepley FJ III, Johnston AD, Kent AJ (2017) Trace element partitioning between plagioclase and melt: an investigation of the impact of experimental and analytical procedures. Geochem Geophys Geosyst 18:3359–3384. 10.1002/2017GC007080

[CR89] Osborne ZR, Thomas JB, Nachlas WO, Angel RJ, Hoff CM, Watson EB (2022) TitaniQ revisited: expanded and improved Ti-in-quartz solubility model for thermobarometry. Contrib Mineral Petrol 177:31. 10.1007/s00410-022-01896-8

[CR90] Pamukcu AS, Ghiorso MS, Gualda GAR (2016) High-Ti, bright-CL rims in volcanic quartz: a result of very rapid growth. Contrib Mineral Petrol 171:105. 10.1007/s00410-016-1317-x

[CR91] Paton C, Hellstrom J, Paul B, Woodhead J, Hergt J (2011) Iolite: freeware for the visualisation and processing of mass spectrometric data. J Anal at Spectrom 26:2508–2518. 10.1039/c1ja10172b

[CR92] Randolph AD, Larson MA (1988) Theory of particulate processes, 2nd edn. Elsevier, New York, pp 109–134

[CR93] Ratschbacher BC, Keller CB, Cooper KM (2024) Insights into magma reservoir dynamics from a global comparison of volcanic and plutonic zircon trace element variability in individual hand samples. Geochem Geophys Geosyst 25:e2024GC011681. 10.1029/2024GC011681

[CR94] Reid MR, Vazquez JA (2017) Fitful and protracted magma assembly leading to a giant eruption, Youngest Toba Tuff, Indonesia. Geochem Geophys Geosyst 18:156–177. 10.1002/2016GC006641

[CR95] Samperton KM, Schoene B, Cottle JM, Keller CB, Crowley JL, Schmitz MD (2015) Magma emplacement, differentiation and cooling in the middle crust: Integrated zircon geochronological–geochemical constraints from the Bergell Intrusion, Central Alps. Chem Geol 417:322–340. 10.1016/j.chemgeo.2015.10.024

[CR96] Schaen AJ, Jicha BR, Hodges KV, Vermeesh P, Stelten ME, Mercer CM, Philipps D, Rivera TA, Jourdan F, Matchan EL, Hemming SR, Morgan LE, Kelley SP, Cassata WS, Heizler MT, Vasconcelos PM, Benowitz JA, Koppers AAP, Mark DF, Niespolo EM, Sprain CJ, Hames WE, Kuiper KF, Turrin BD, Renne PR, Ross J, Nomade S, Guillou H, Webb LE, Cohen BA, Calvert AT, Joyce N, Ganerød M, Wijbrans J, Ishizuka O, He H, Ramirez A, Pfänder JA, Lopez-Martínez M, Qiu H, Singer BS (2020) Interpreting and reporting ^40^Ar/^39^Ar geochronologic data. Geol Soc Am Bull 133:461–487. 10.1130/B35560.1

[CR97] Schaen AJ, Schoene B, Dufek J, Singer BS, Eddy MP, Jicha BR, Cottle JM (2021) Transient rhyolite melt extraction to produce a shallow granitic pluton. Sci Adv. 7:eabf0604. 10.1126/sciadv.abf0604 34138741 10.1126/sciadv.abf0604 PMC8133745

[CR98] Schaltegger U, Brack P, Ovtcharova M, Peytcheva I, Schoene B, Stracke A, Marocchi M, Bargossi GM (2009) Zircon and titanite recording 1.5 million years of magma accretion, crystallization and initial cooling in a composite pluton (southern Adamello batholith, northern Italy). Earth Planet Sci Lett 286:208–218. 10.1016/j.epsl.2009.06.028

[CR99] Schaltegger U, Nowak A, Ulianov A, Fisher CM, Gerdes A, Spikings R, Whitehouse MJ, Bindeman IN, Hanchar JM, Duff J, Vervoort JD, Sheldrake T, Caricchi L, Brack P, Müntener O (2019) Zircon petrochronology and ^40^Ar/^39^Ar thermochronology of the Adamello Intrusive Suite, N. Italy: Monitoring the growth and decay of an incrementally assembled magmatic system. J Petrol 60:701–722. 10.1093/petrology/egz010

[CR100] Schlieder TD, Cooper KM, Kent AJ, Bradshaw R, Huber C (2022) Thermal storage conditions and origin of compositional diversity of plagioclase crystals in magmas from the 1980 and 2004–2005 eruptions of Mount Saint Helens. J Petrol 63:egac064. 10.1093/petrology/egac064

[CR101] Schoene B, Schaltegger U, Brack P, Latkoczy C, Stracke A, Günther D (2012) Rates of magma differentiation and emplacement in a ballooning pluton recorded by U-Pb TIMS-TEA, Adamello batholith, Italy. Earth Planet Sci Lett 355:162–173. 10.1016/j.epsl.2012.08.019

[CR102] Shamloo HI, Till CB (2019) Decadal transition from quiescence to supereruption: petrologic investigation of the Lava Creek Tuff, Yellowstone Caldera. WY Contrib Mineral Petrol 174:32. 10.1007/s00410-019-1570-x

[CR103] Sparks RSJ, Sigurdsson H, Wilson L (1977) Magma mixing: a mechanism for triggering acid explosive eruptions. Nature 267:315–318. 10.1038/267315a0

[CR104] Spear FS, Parrish RR (1996) Petrology and cooling rates of the Valhalla complex, British Columbia, Canada. J Petrol 37:733–765. 10.1093/petrology/37.4.733

[CR105] Swanson SE (1977) Relation of nucleation and crystal–growth rate to the development of granitic textures. Am Min 62:966–978

[CR106] Szymanowski D, Forni F, Phua M, Jicha B, Lee DW, Hsu YJ, Rifai H, Schoene B, de Maisonneuve CB (2023) A shifty Toba magma reservoir: Improved eruption chronology and petrochronological evidence for lateral growth of a giant magma body. Earth Planet Sci Lett 622:118408. 10.1016/j.epsl.2023.118408

[CR107] Tavazzani L, Peres S, Sinigoi S, Demarchi G, Economos RC, Quick JE (2020) Timescales and mechanisms of crystal–mush rejuvenation and melt extraction recorded in Permian plutonic and volcanic rocks of the Sesia Magmatic System (southern Alps, Italy). J Petrol 61:egaa049. 10.1093/petrology/egaa049

[CR108] Tavazzani L, Wotzlaw JF, Economos RC, Sinigoi S, Demarchi G, Szymanowski D, Laurent O, Bachmann O, Chelle-Michou C (2023) High-precision zircon age spectra record the dynamics and evolution of large open-system silicic magma reservoirs. Earth Planet Sci Lett 623:118432. 10.1016/j.epsl.2023.118432

[CR109] Thomas JB, Watson EB, Spear FS, Shemella PT, Nayak SK, Lanzirotti A (2010) TitaniQ under pressure: the effect of pressure and temperature on the solubility of Ti in quartz. Contrib Mineral Petrol 160:743–759. 10.1007/s00410-010-0505-3

[CR110] Thomas JB, Watson EB, Spear FS, Wark DA (2015) TitaniQ recrystallized: experimental confirmation of the original Ti-in-quartz calibrations. Contrib Mineral Petrol 169:1–16. 10.1007/s00410-015-1120-0

[CR111] Till CB, Vazquez JA, Boyce JW (2015) Months between rejuvenation and volcanic eruption at Yellowstone caldera, Wyoming. Geology 43:695–698. 10.1130/G36862.1

[CR112] Ulmer P, Callegari E, Sonderegger UC (1983) Genesis of the mafic and ultramafic rocks and their genetical relations to the tonalitic-trondhjemitic granitoids of the southern part of the Adamello batholith (northern Italy). Mem Soc Geol Italiana 26:171–222

[CR113] Van Orman JA, Cherniak DJ, Kita NT (2014) Magnesium diffusion in plagioclase: dependence on composition, and implications for thermal resetting of the ^26^Al–^26^Mg early solar system chronometer. Earth Planet Sci Lett. 385:79–88. 10.1016/j.epsl.2013.10.026

[CR114] Vazquez JA, Reid MR (2004) Probing the accumulation history of the voluminous Toba magma. Science 305:991–994. 10.1126/science.1096994 15310899 10.1126/science.1096994

[CR115] Verberne R (2013) The role of magma rheology during emplacement of the Listino Suite, Adamello Massif, Italy. PhD thesis, University of Lausanne.

[CR116] Walker BA Jr, Miller CF, Claiborne LL, Wooden JL, Miller JS (2007) Geology and geochronology of the Spirit Mountain batholith, southern Nevada: Implications for timescales and physical processes of batholith construction. J Volcanol Geotherm Res 167:239–262. 10.1016/j.jvolgeores.2006.12.008

[CR117] Wang DB, Liu PP, Gao MH, Zhang D, Xu C, Caricchi L (2023) Prolonged near-solidus and steady-state magma storage for the Youngest Toba Tuff: evidence from TitaniQ thermometry and diffusion chronometry. Earth Planet Sci Lett 619:118326. 10.1016/j.epsl.2023.118326

[CR118] Wark DA, Spear FS (2005) Ti in quartz: cathodoluminescence and thermometry. Geochim Cosmochim Acta Suppl 69:A592

[CR119] Wark DA, Watson EB (2006) TitaniQ: a titanium-in-quartz geothermometer. Contrib Mineral Petrol 152:743–754. 10.1007/s00410-006-0132-1

[CR120] Wark DA, Hildreth W, Spear FS, Cherniak DJ, Watson EB (2007) Pre-eruption recharge of the Bishop magma system. Geology 35:235–238. 10.1130/G23316A.1

[CR121] Weber G, Caricchi L, Arce JL, Schmitt AK (2020) Determining the current size and state of subvolcanic magma reservoirs. Nat Commun 11:5477–5414. 10.1038/s41467-020-19084-2 33154361 10.1038/s41467-020-19084-2 PMC7644707

[CR122] Wotzlaw JF, Schaltegger U, Frick DA, Dungan MA, Gerdes A, Günther D (2013) Tracking the evolution of large-volume silicic magma reservoirs from assembly to supereruption. Geology 41:867–870. 10.1130/G34366.1

[CR123] Wotzlaw JF, Bindeman IN, Watts KE, Schmitt AK, Caricchi L, Schaltegger U (2014) Linking rapid magma reservoir assembly and eruption trigger mechanisms at evolved Yellowstone-type supervolcanoes. Geology 42:807–810. 10.1130/G35979.1

[CR124] Yoder HS, Stewart DB, Smith JR (1957) Ternary Feldspars. Carnegie Inst Washington Yearbook 56:206–214

[CR125] Zhang C, Li X, Almeev RR, Horn I, Behrens H, Holtz F (2020) Ti-in-quartz thermobarometry and TiO_2_ solubility in rhyolitic melts: new experiments and parametrization. Earth Planet Sci Lett 538:116213. 10.1016/j.epsl.2020.116213

