# Supplementary material for: Cooling rates and melt extraction timescales determined by diffusion chronometry on shallow crustal plutonic rocks
Source: Contrib Mineral Petrol. 2025 Jul 11;180(8):45. doi: 10.1007/s00410-025-02238-0 (PMC12254178; doi:10.1007/s00410-025-02238-0)
Supplement: Supplementary file 1 — Supplementary file1 (PDF 4836 KB) [file 410_2025_2238_MOESM1_ESM.pdf]

Electronic Supplementary Material 1 for

Cooling rates and melt extraction timescales determined by diffusion chronometry in shallow crustal plutonic rocks

Thomas Grocolas[1*], Othmar Müntener[1], Elias M. Bloch[1,2], Stéphane Escrig[1,3], Alexey Ulyanov[1], Anne-Sophie Bouvier[1]

[1]Institute of Earth Sciences, University of Lausanne, Géopolis, CH-1015 Lausanne, Switzerland

[2]Lunar and Planetary Laboratory, University of Arizona, Tucson, AZ 85721, USA

[3]Laboratory for Biological Geochemistry, School of Architecture, Civil and Environmental Engineering, Ecole Polytechnique Fédérale de Lausanne, CH-1015 Lausanne, Switzerland

[*]Corresponding author; E-mail address: thomas.grocolas@unil.ch

Information

The Electronic Supplementary Material 1 (ESM 1) contains (1) a Supplementary Text detailing the calculations of the uncertainties related to analysis and to diffusion modelling, and of the thermal modelling, and (2) Supplementary Figures highlighting the measured plagioclase and amphibole compositions, the fitted plagioclase and quartz compositional profiles used to determine cooling rates and residence times, and a sketch explaining the design of the thermal model.

Supplementary Text 1. Analysis and model uncertainties.

Analytical uncertainties using LA-ICP-MS are mainly associated with counting statistics, standardisation, and the long-term reproducibility, often referred to as "systematic uncertainty." For raster measurements, uncertainties were determined by calculating the relative standard deviation of the measured abundances obtained from the analysis of BCR-2G from its known abundances (Jochum et al., 2011). The typical standard deviation is in the range of ∼2-6 % (2 S.D.) for Sr and Ba. The systematic uncertainty, which accounts for the long-term scattering of the mean value relative to the known abundance, was not considered in this case, but is estimated to be <5 % (2 S.D.).
A similar operation was done to calculate the uncertainties on SIMS measurements, but compositional variations between acquisition cycles were also considered, and errors were propagated to give typical uncertainties for Ti concentrations in quartz of ∼2-3 % (2 S.D.).
For NanoSIMS, the high-resolution ^{48}Ti/^{28}Ti profiles in quartz and Sr profiles in plagioclase are reported as cumulated counts. As such, analytical uncertainties only depend on the counting statistics and are typically associated with a relative standard deviation of <3 % (2 S.D.) for the ^{48}Ti/^{28}Si ratio. Such uncertainty is lower than the symbol size and is therefore not reported in Fig. 7 from the main text and in Supplementary Figure 5.

Several parameters used during diffusion modelling are associated with an uncertainty which must be considered when calculating timescales. In addition to the initial profile, the uncertainty budget is largely controlled by the initial temperature, the parameters of the diffusion coefficients ($\log_{10} D_0$ and E_a), and the curve fitting including the measurement uncertainties (Costa et al., 2008; Wu et al., 2022). In this study, we only consider uncertainties related to the geometry of the initial profile (for quartz), temperature, and diffusion coefficients. In normally-zoned crystals, the impact of crystal growth on the initial conditions is probably more important than the uncertainty on the curve fitting, especially when the profile shape is well-resolved. To evaluate the total uncertainty, a Monte Carlo approach was employed for each profile where 1,500 trials were performed. The uncertainty structure of $\log_{10} D_0$ and E_a is strongly covariant (Mutch et al., 2021; Wu et al., 2022) and must be considered during the resampling process, which otherwise would lead to overestimated total uncertainties. For this model, we assume that $\log_{10} D_0$ and E_a follow a linear trend without any uncertainty envelope, which slightly underestimate the total uncertainty. For modelling cooling rates, the initial temperature and diffusion parameters were sampled following a Gaussian distribution around the mean, and profiles were modelled using Eqs. 2 and 5 from the main text, and fitted with a least-square optimisation routine. In addition to the abovementioned parameters, the calculated crystal-melt segregation timescales also account for the uncertainties associated with the cooling rates. The Monte Carlo model

randomly samples the exponential factor controlling the cooling rate around the mean and considers a standard deviation of 20 %. The uncertainties related to the calculated cooling rates and crystal-melt segregation timescales are reported as the two-fold standard deviation.

Supplementary Text 2. Thermal modelling.

The temperature evolution of the Western Adamello tonalite was modelled by solving the two-dimensional axisymmetric formulation of the heat equation (Eq. 1):

$$\rho c \frac{\partial T}{\partial t} = \frac{1}{r}\frac{\partial}{\partial r}\left(rk\frac{\partial T}{\partial r}\right) + \frac{\partial}{\partial z}\left(k\frac{\partial T}{\partial z}\right) + \rho L\left(\frac{\partial X_{\mathrm{c}}}{\partial t}\right), \tag{1}$$

where T is the temperature (°C), t is the time (s), r is the radial coordinate relative to the symmetry axis (m), z is the depth (m), k is the thermal conductivity (W m^{-1} K^{-1}), L is the latent heat of crystallisation (J kg^{-1}), ρ is the magma density (kg m^{-3}), c is the specific heat (J kg^{-1} K^{-1}), and X_{c} is the magma crystallinity. The values associated with these parameters can be found in the Electronic Supplementary Material ESM 2. The employed liquidus and solidus temperatures were 950 °C and 670 °C, respectively, while the magma crystallinity was parameterised based on the experiments of Marxer and Ulmer (2019) and follows Eq. 2:

$$X_{\mathrm{c}} = -0.002632 \times T + 2.7105. \tag{2}$$

Calculations were performed based on those of Floess and Baumgartner (2015), whereby a magma reservoir grows by the horizontal stacking of vertical, 20-m-thick dikes with a flow time of 140 yr and a no-flow time of 2,200 yr. These values correspond to an average magma flux of 2.5×10^{-4} km^3 yr^{-1}, assuming a dike length of 3 km and a width of 10 km, which is similar to the flux inferred from high-precision zircon dating (Floess, 2013).

Eq. 1 was numerically solved using an explicit finite-difference method. The numerical grid was large enough (15 × 15 km) so that the boundary conditions were kept constant. The temperature evolution, as shown on Fig. 10 from the main text, was tracked at a point located 2 km away from the southwestern contact, which corresponds to the average of the three investigated leucotonalite samples. Following the high-precision zircon ages (Floess, 2013), the magma flux was stopped after 1.2 Myr of dike injection. Although this ignores the subsequent development of the Central Adamello leucotonalite (CAL), the leucotonalite sample are located ≥ 1 km away from the contact with the CAL, therefore limiting its thermal impact on the central part of the WAT. The complete temperature evolution of the point of interest can be found in Supplementary Figure 7.

Supplementary Figure 1. Major and trace element bulk rock compositions of the Western Adamello leucotonalite and Laione granodiorite. **a**, Total alkali silica [SiO_2 (wt.%) vs. $Na_2O + K_2O$ (wt.%)] diagram (Middlemost, 1994). **b** SiO_2 (wt.%) vs. Sr (µg/g). **c** SiO_2 (wt.%) vs. Ba (µg/g). **d** SiO_2 (wt.%) vs. Eu/Eu*. The larger symbols correspond to the samples investigated in this study, and the smaller symbols represent other samples from the same lithologies. The data from the Adamello batholith are from the compilation of Müntener et al. (2021).

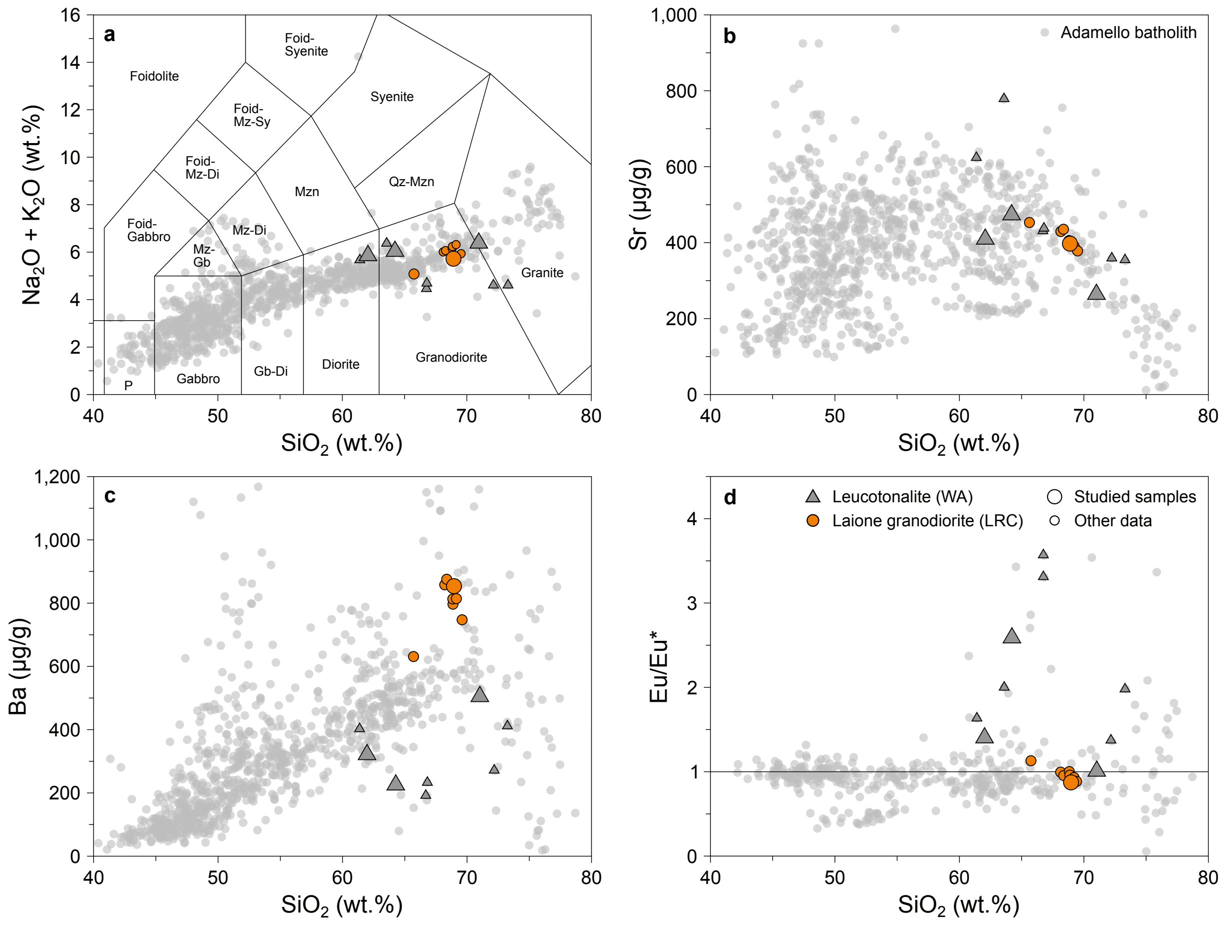

Supplementary Figure 2. Composition of plagioclase from the Listino ring complex measured by LA-ICP-MS spot analysis. **a**, An (mol.%) vs. Sr (µg/g). **b**, An (mol.%) vs. Ba (µg/g). Core-rim pairs are shown to highlight the slope and, therefore, the mechanism controlling trace element diffusion in plagioclase.

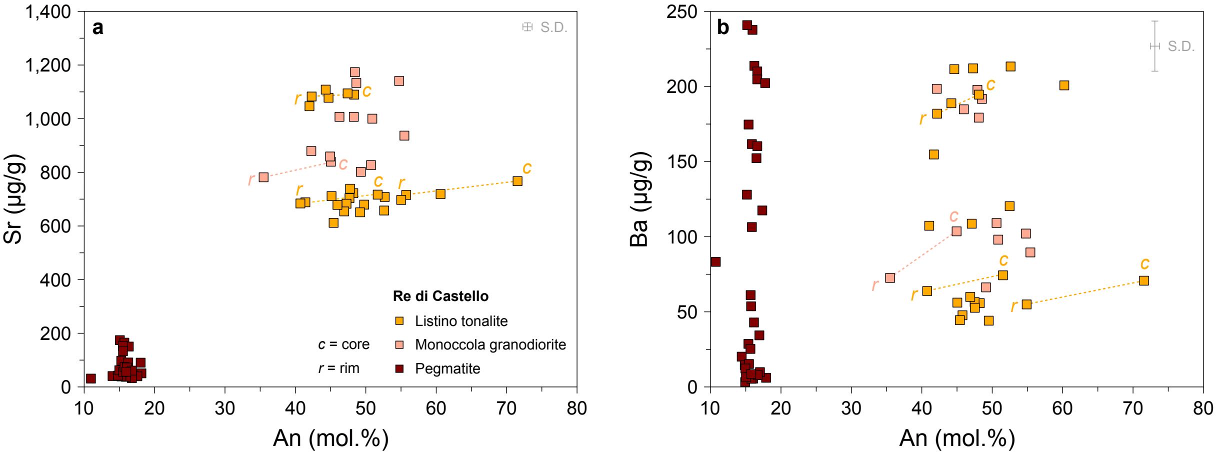

Supplementary Figure 3. Composition of amphibole from the Re di Castello superunit, which comprised the Listino ring complex, plotted as Al^{IV} (a.p.f.u.) vs. $(Na + K)^{A}$ (a.p.f.u.). The edenite exchange controlling the amphibole chemistry is also represented.

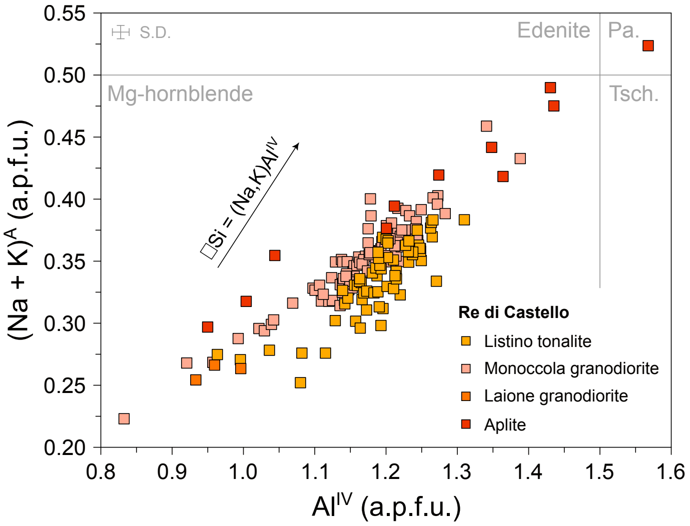

Supplementary Figure 4. Measured profiles and results of Sr- and Ba-in-plagioclase diffusion modelling used to infer cooling rates. The black dotted line represents the initial conditions, the red line corresponds to the best fit to the data (blue), and the yellow data points are for the calculated quasi-steady state profile. The diagrams highlighting the temperature (°C) evolution through time (Myr) show the best fits using different diffusion coefficients for Sr and Ba diffusion in plagioclase (Cherniak and Watson, 1992, 1994; Cherniak, 2002; Giletti and Casserly, 1994; Grocolas et al., 2025). The $^{39}Ar/^{40}Ar$ ages obtained on minerals with different closure temperatures (Schaltegger et al., 2019) are represented for comparison.

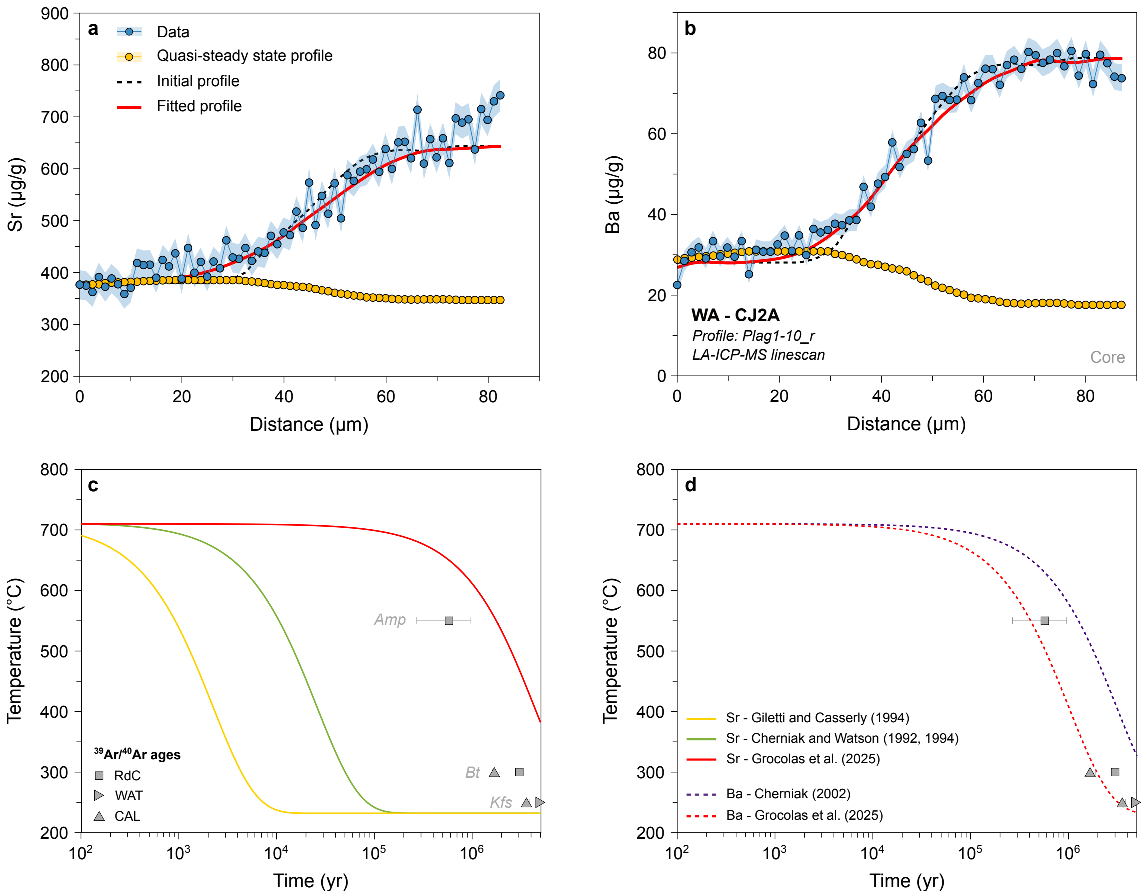

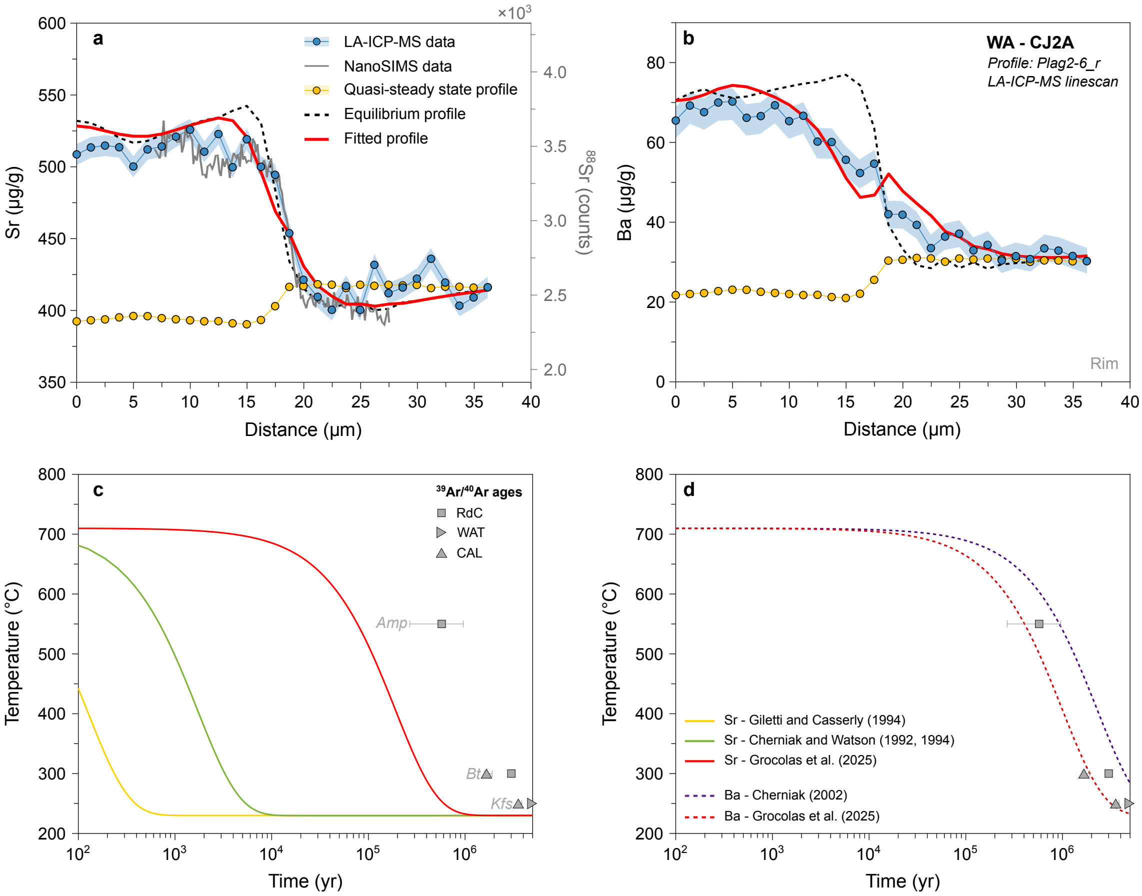
a
LA-ICP-MS data
NanoSIMS data
Quasi-steady state profile
Equilibrium profile
Fitted profile
Sr (µg/g)
^{88}Sr (counts)
×10^3
Distance (µm)
b
WA - CJ2A
Profile: Plag2-6_r
LA-ICP-MS linescan
Ba (µg/g)
Rim
Distance (µm)
c
^{39}Ar/^{40}Ar ages
RdC
WAT
CAL
Amp
Bt
Kfs
Temperature (°C)
Time (yr)
d
Sr - Giletti and Casserly (1994)
Sr - Cherniak and Watson (1992, 1994)
Sr - Grocolas et al. (2025)
Ba - Cherniak (2002)
Ba - Grocolas et al. (2025)
Temperature (°C)
Time (yr)

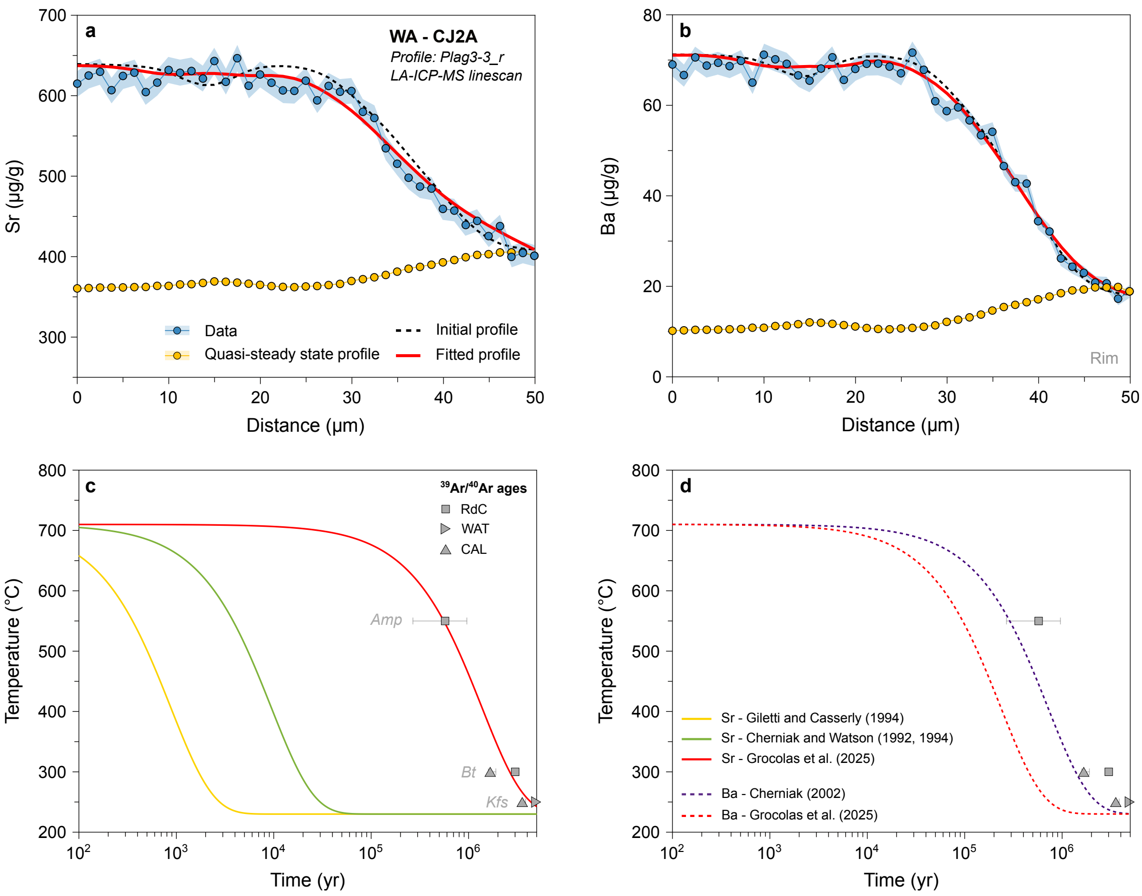
a
WA - CJ2A
Profile: Plag3-3_r
LA-ICP-MS linescan
Sr (µg/g)
Data
Quasi-steady state profile
Initial profile
Fitted profile
Distance (µm)
b
Ba (µg/g)
Rim
Distance (µm)
c
^{39}Ar/^{40}Ar ages
RdC
WAT
CAL
Amp
Bt
Kfs
Temperature (°C)
Time (yr)
d
Sr - Giletti and Casserly (1994)
Sr - Cherniak and Watson (1992, 1994)
Sr - Grocolas et al. (2025)
Ba - Cherniak (2002)
Ba - Grocolas et al. (2025)
Temperature (°C)
Time (yr)

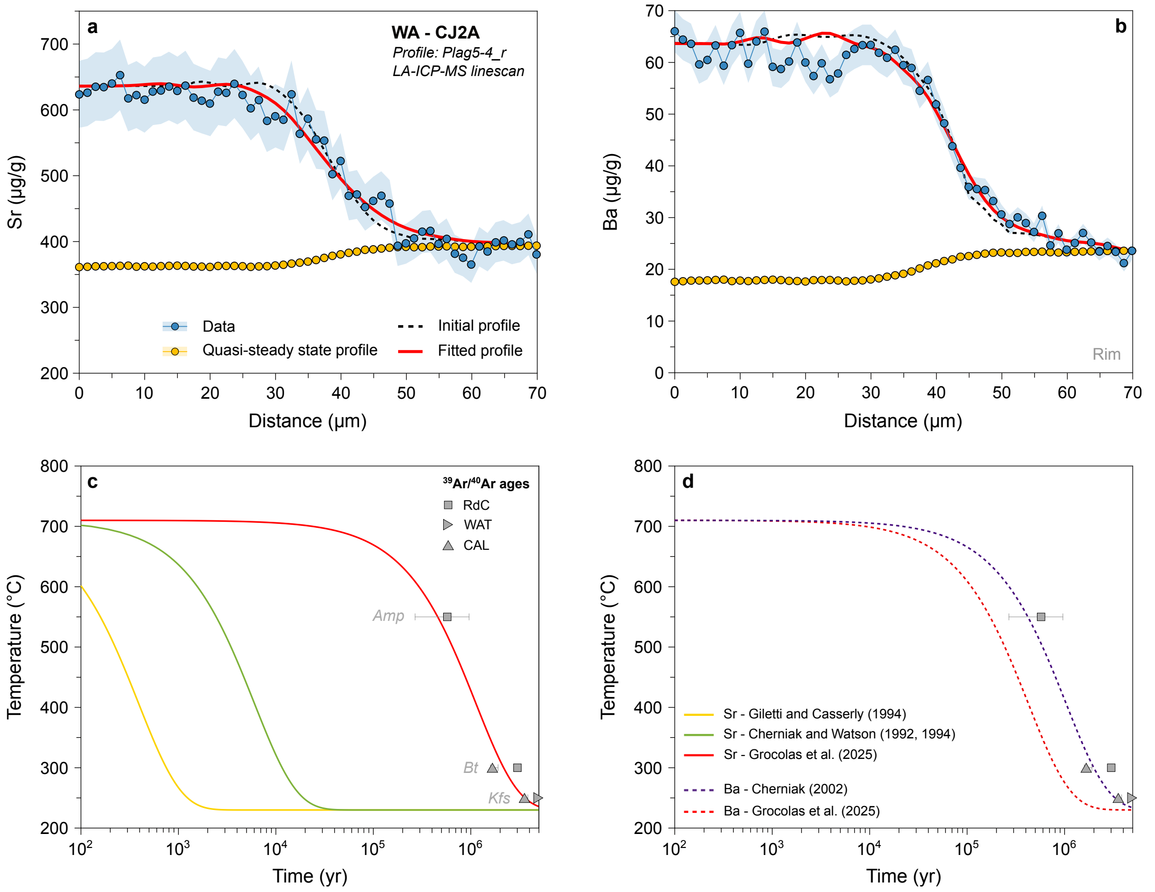
a
WA - CJ2A
Profile: Plag5-4_r
LA-ICP-MS linescan
Sr (µg/g)
Distance (µm)
Data
Quasi-steady state profile
Initial profile
Fitted profile
b
Ba (µg/g)
Rim
Distance (µm)
c
39Ar/40Ar ages
RdC
WAT
CAL
Amp
Bt
Kfs
Temperature (°C)
Time (yr)
d
Sr - Giletti and Casserly (1994)
Sr - Cherniak and Watson (1992, 1994)
Sr - Grocolas et al. (2025)
Ba - Cherniak (2002)
Ba - Grocolas et al. (2025)
Temperature (°C)
Time (yr)

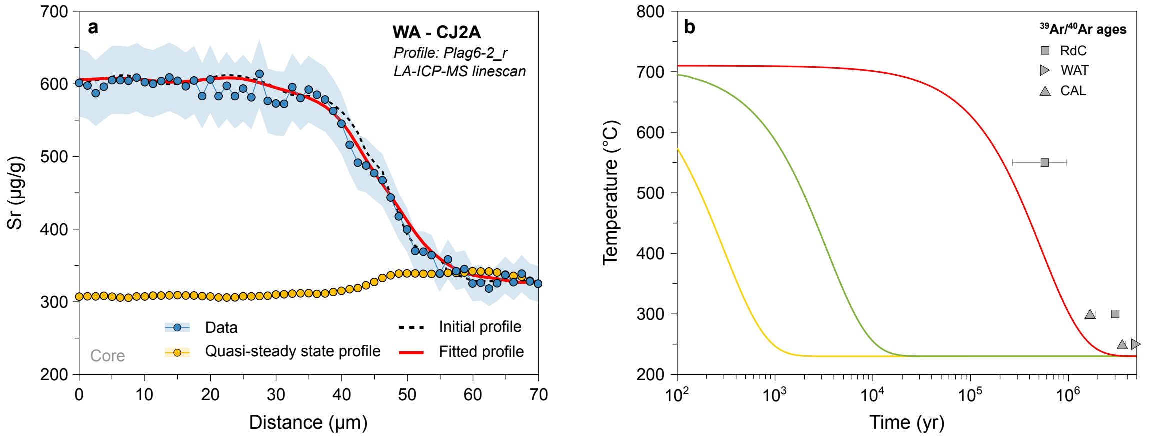
a
WA - CJ2A
Profile: Plag6-2_r
LA-ICP-MS linescan
Sr (µg/g)
Core
Data
Quasi-steady state profile
Initial profile
Fitted profile
Distance (µm)
b
39Ar/40Ar ages
RdC
WAT
CAL
Temperature (°C)
Time (yr)

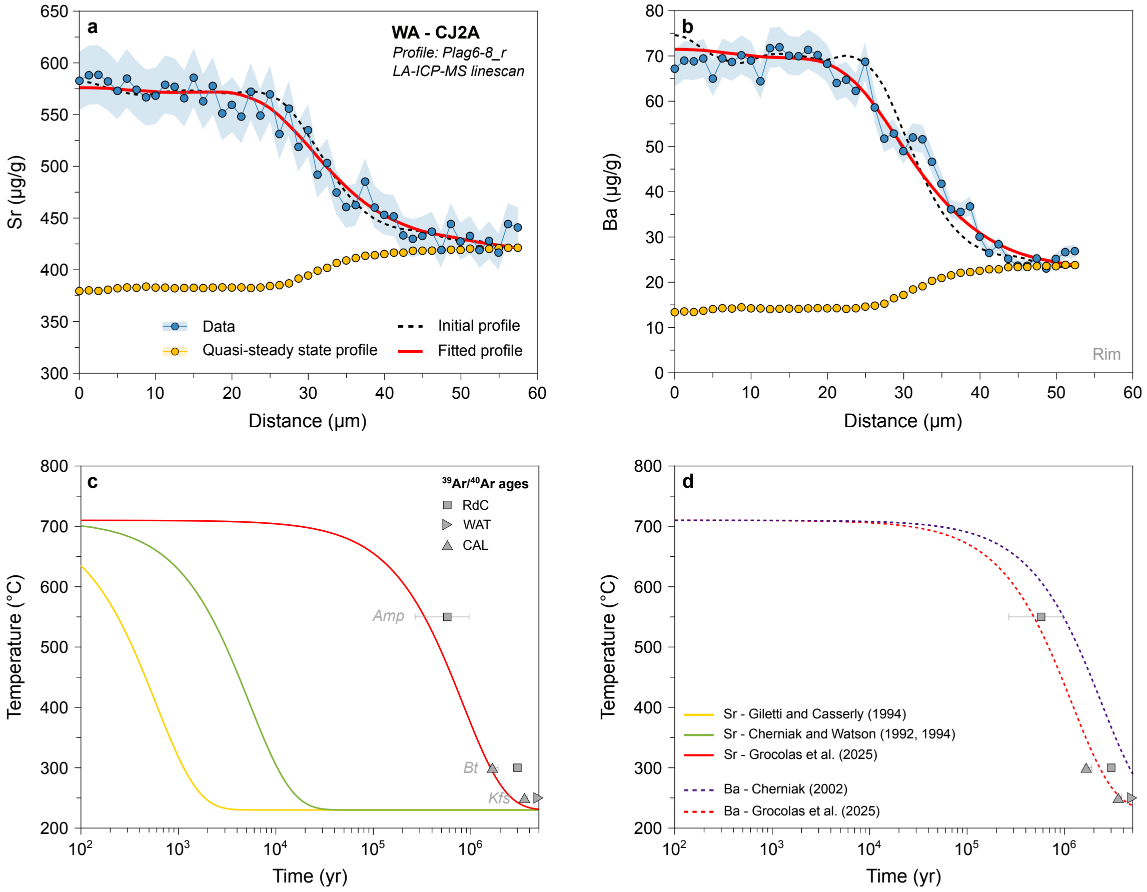
a
WA - CJ2A
Profile: Plag6-8_r
LA-ICP-MS linescan
Sr (µg/g)
Distance (µm)
Data
Quasi-steady state profile
Initial profile
Fitted profile
b
Ba (µg/g)
Rim
Distance (µm)
c
^{39}Ar/^{40}Ar ages
RdC
WAT
CAL
Amp
Bt
Kfs
Temperature (°C)
Time (yr)
d
Sr - Giletti and Casserly (1994)
Sr - Cherniak and Watson (1992, 1994)
Sr - Grocolas et al. (2025)
Ba - Cherniak (2002)
Ba - Grocolas et al. (2025)
Temperature (°C)
Time (yr)

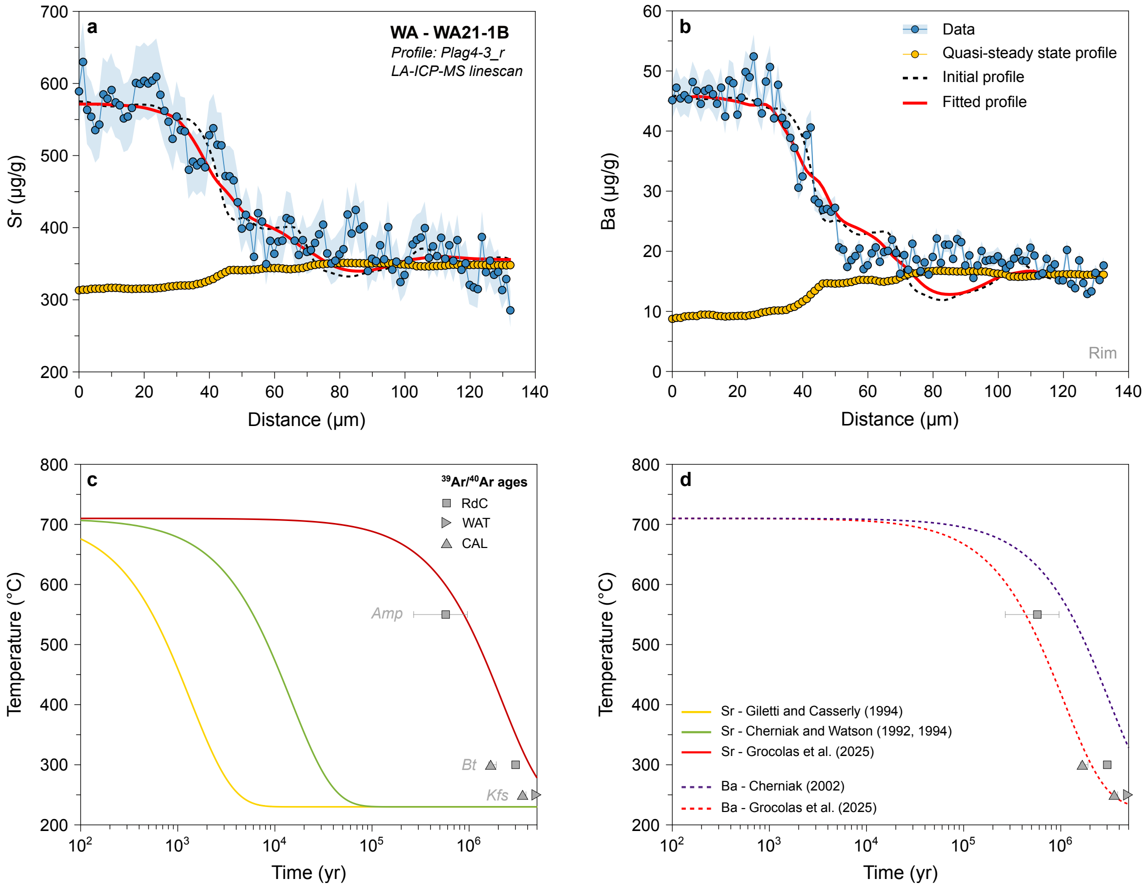
a
WA - WA21-1B
Profile: Plag4-3_r
LA-ICP-MS linescan
Sr (µg/g)
Distance (µm)
b
Data
Quasi-steady state profile
Initial profile
Fitted profile
Ba (µg/g)
Rim
Distance (µm)
c
^{39}Ar/^{40}Ar ages
RdC
WAT
CAL
Amp
Bt
Kfs
Temperature (°C)
Time (yr)
d
Sr - Giletti and Casserly (1994)
Sr - Cherniak and Watson (1992, 1994)
Sr - Grocolas et al. (2025)
Ba - Cherniak (2002)
Ba - Grocolas et al. (2025)
Temperature (°C)
Time (yr)

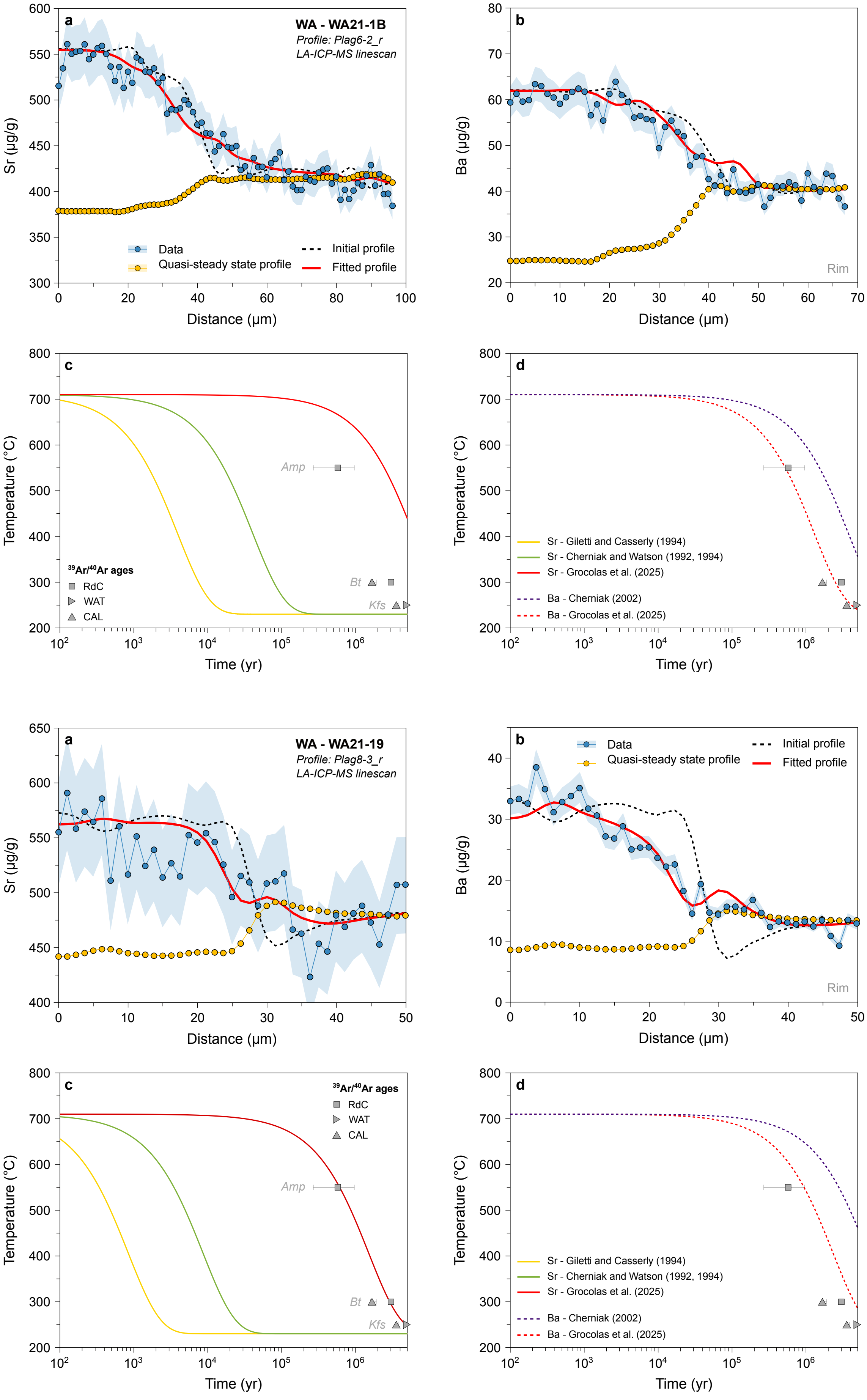
a
WA - WA21-1B
Profile: Plag6-2_r
LA-ICP-MS linescan
Sr (µg/g)
Distance (µm)
Data
Quasi-steady state profile
Initial profile
Fitted profile
b
Ba (µg/g)
Rim
Distance (µm)
c
Temperature (°C)
Time (yr)
Amp
Bt
Kfs
39Ar/40Ar ages
RdC
WAT
CAL
d
Sr - Giletti and Casserly (1994)
Sr - Cherniak and Watson (1992, 1994)
Sr - Grocolas et al. (2025)
Ba - Cherniak (2002)
Ba - Grocolas et al. (2025)
a
WA - WA21-19
Profile: Plag8-3_r
LA-ICP-MS linescan
b
c
d

Supplementary Figure 5. Results of Ti-in-quartz diffusion modelling used to infer cooling rates. The black dotted line and continuous line represent the two initial conditions tested in the model, and the red line corresponds to the best fit to the data (grey). The diagrams highlighting the temperature (°C) evolution through time (Myr) show the best fits using the two different initial conditions. The $^{39}Ar/^{40}Ar$ ages obtained on minerals with different closure temperatures (Schaltegger et al., 2019) are represented for comparison.

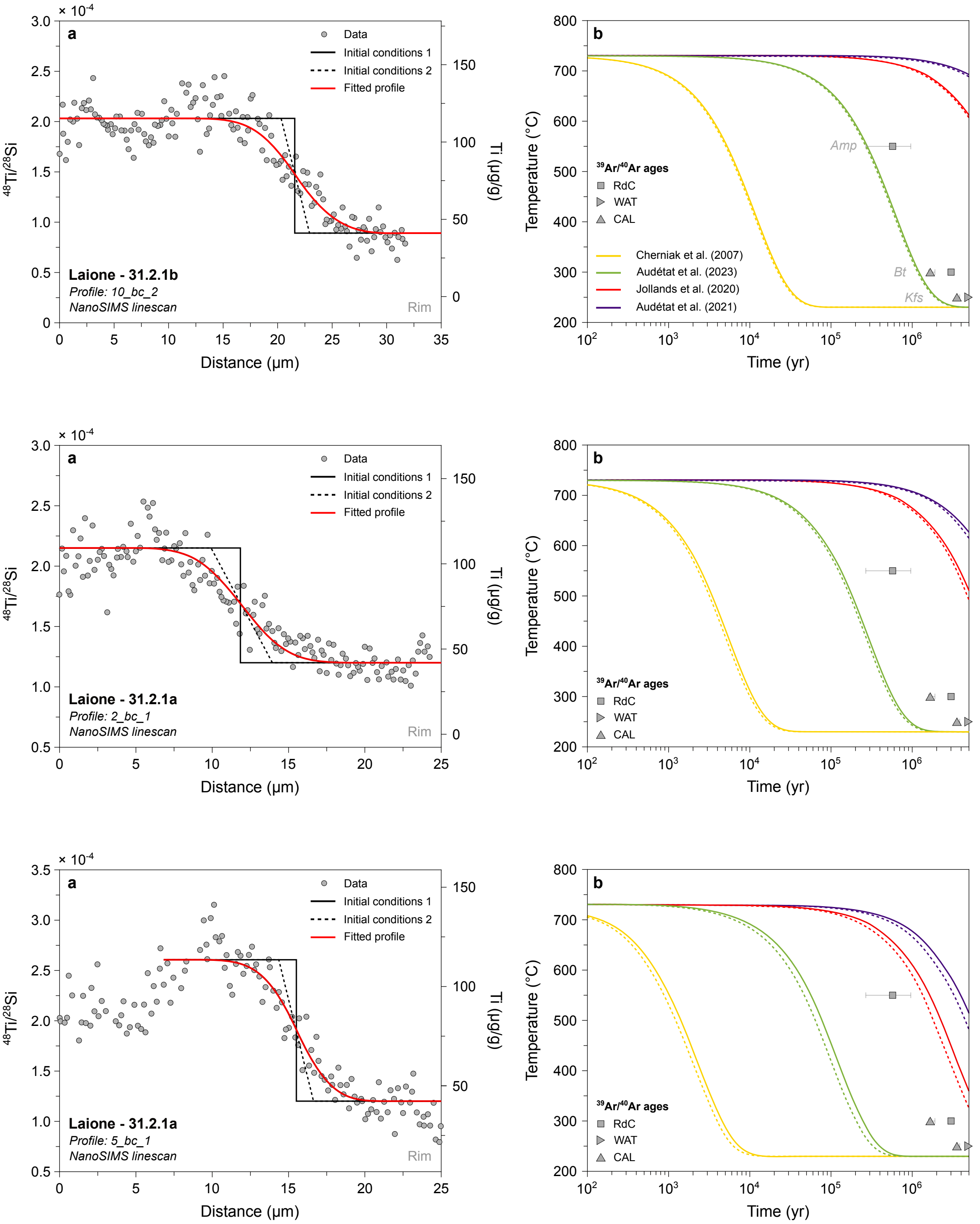

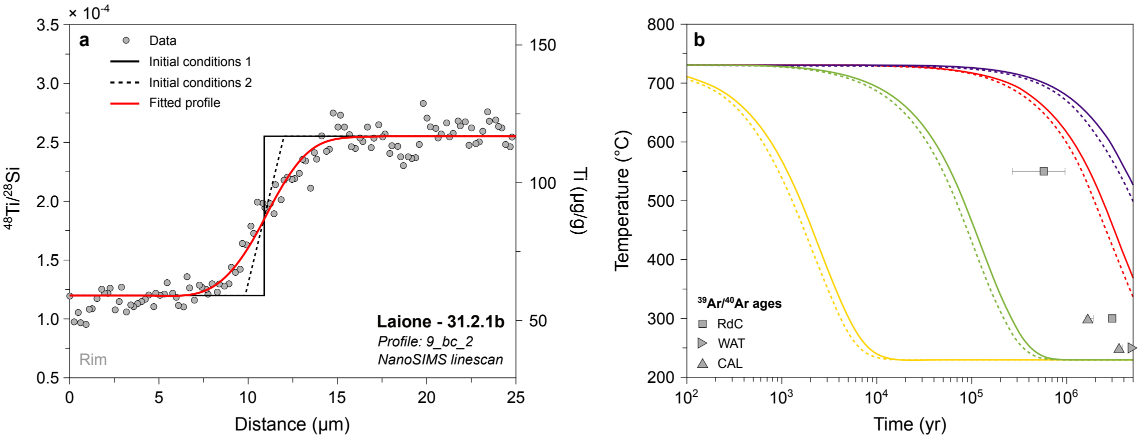
a
b
× 10-4
Data
Initial conditions 1
Initial conditions 2
Fitted profile
48Ti/28Si
Ti (µg/g)
Laione - 31.2.1b
Profile: 9_bc_2
NanoSIMS linescan
Rim
Distance (µm)
Temperature (°C)
Time (yr)
39Ar/40Ar ages
RdC
WAT
CAL

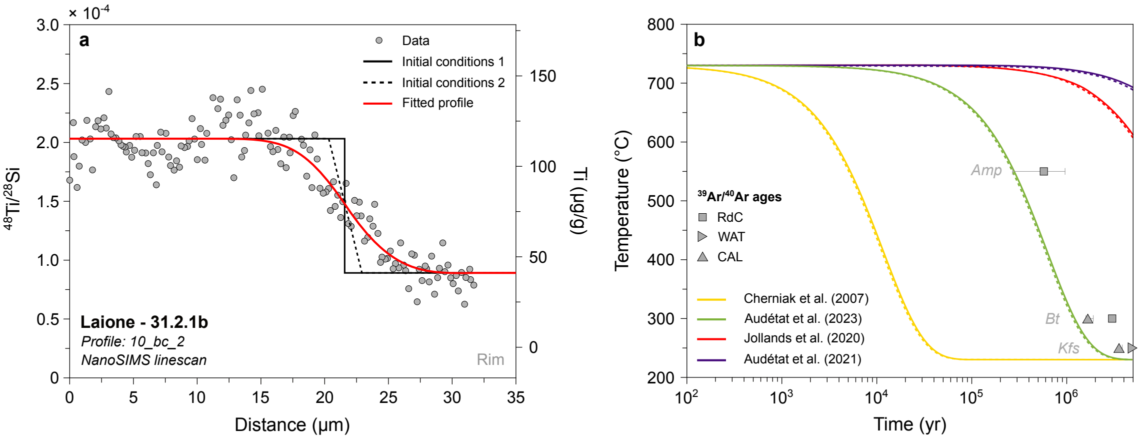
a
b
× 10-4
Data
Initial conditions 1
Initial conditions 2
Fitted profile
48Ti/28Si
Ti (µg/g)
Laione - 31.2.1b
Profile: 10_bc_2
NanoSIMS linescan
Rim
Distance (µm)
Temperature (°C)
Time (yr)
Amp
Bt
Kfs
39Ar/40Ar ages
RdC
WAT
CAL
Cherniak et al. (2007)
Audétat et al. (2023)
Jollands et al. (2020)
Audétat et al. (2021)

Supplementary Figure 6. Results of Sr- and Ba-in-plagioclase diffusion modelling used to infer crystal-melt segregation timescales. The black dotted line represents the initial conditions, the red line corresponds to the best fit to the data (blue), and the yellow data points are for the calculated quasi-steady state profile. The results of the Monte Carlo simulation for Sr and Ba diffusion are represented as temperature (°C) vs. time (kyr). See main text for explanations regarding diffusion modelling and Monte Carlo resampling.

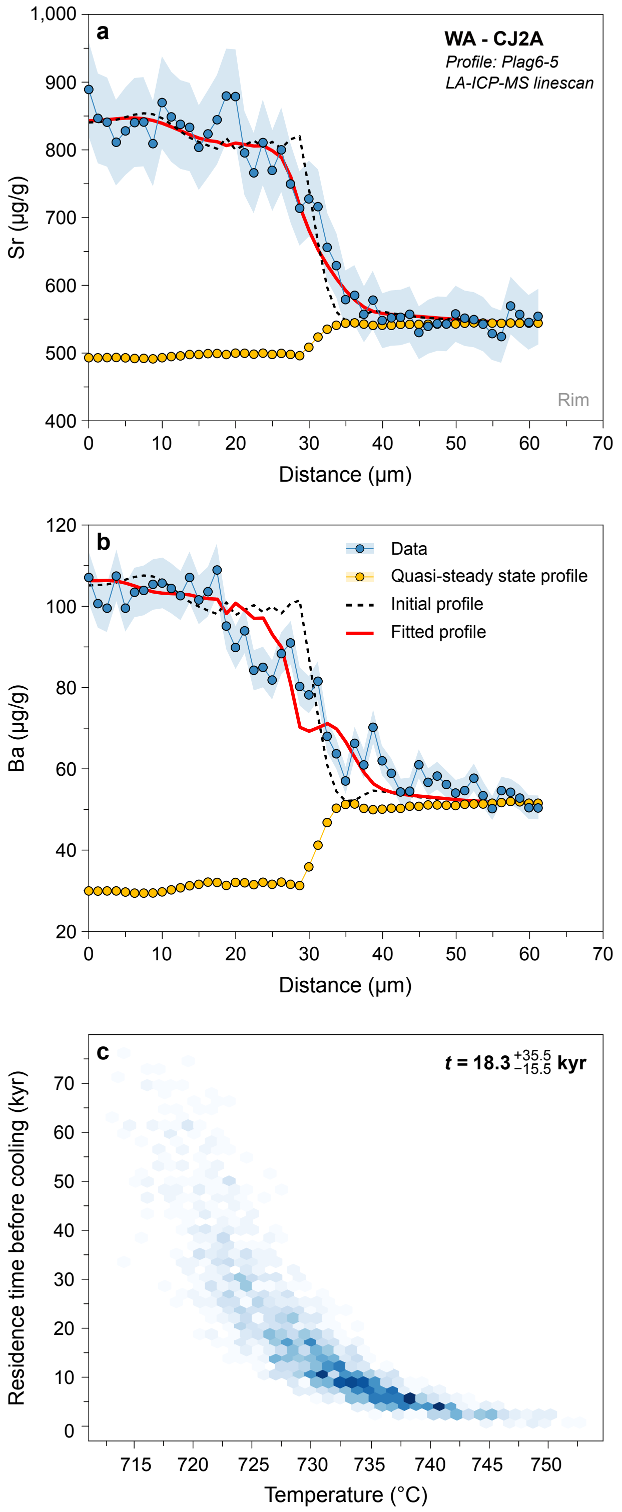

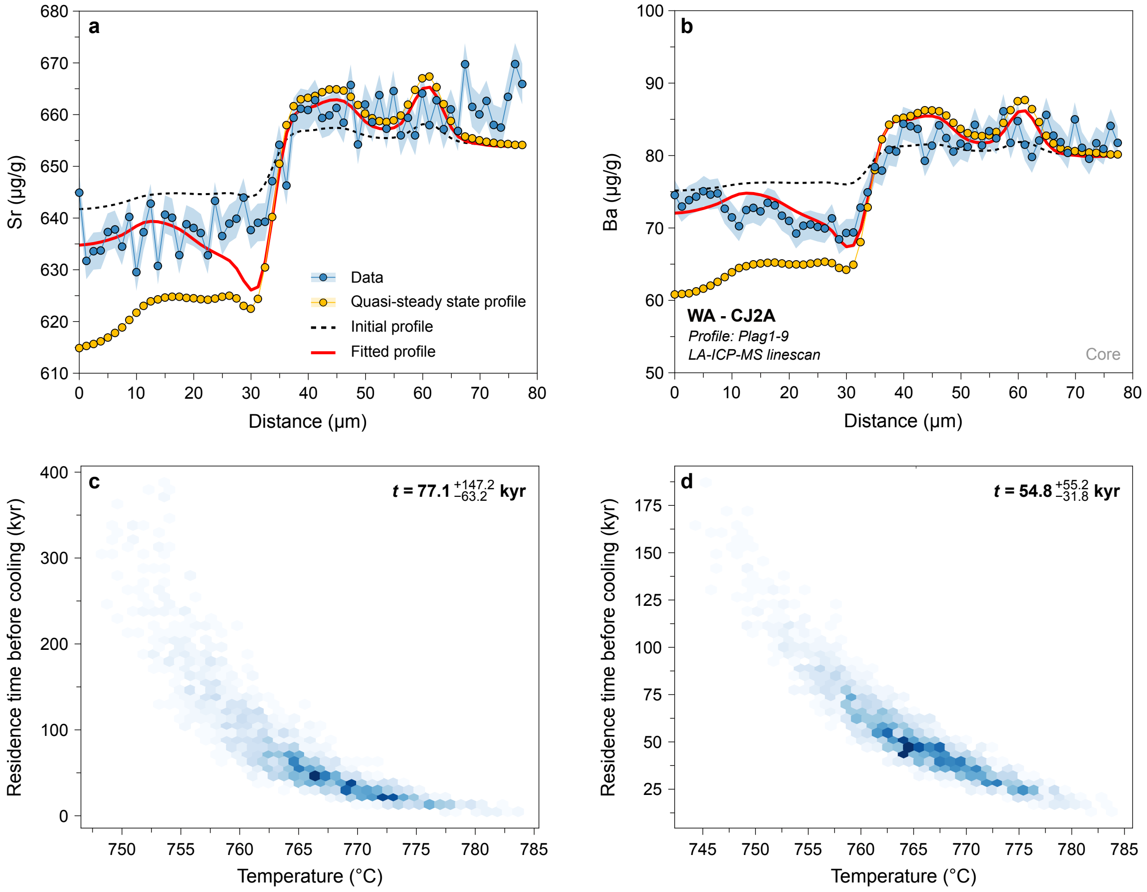
a
Sr (μg/g)
Distance (μm)
Data
Quasi-steady state profile
Initial profile
Fitted profile
b
Ba (μg/g)
WA - CJ2A
Profile: Plag1-9
LA-ICP-MS linescan
Core
c
Residence time before cooling (kyr)
Temperature (°C)
$t = 77.1^{+147.2}_{-63.2}$ kyr
d
$t = 54.8^{+55.2}_{-31.8}$ kyr

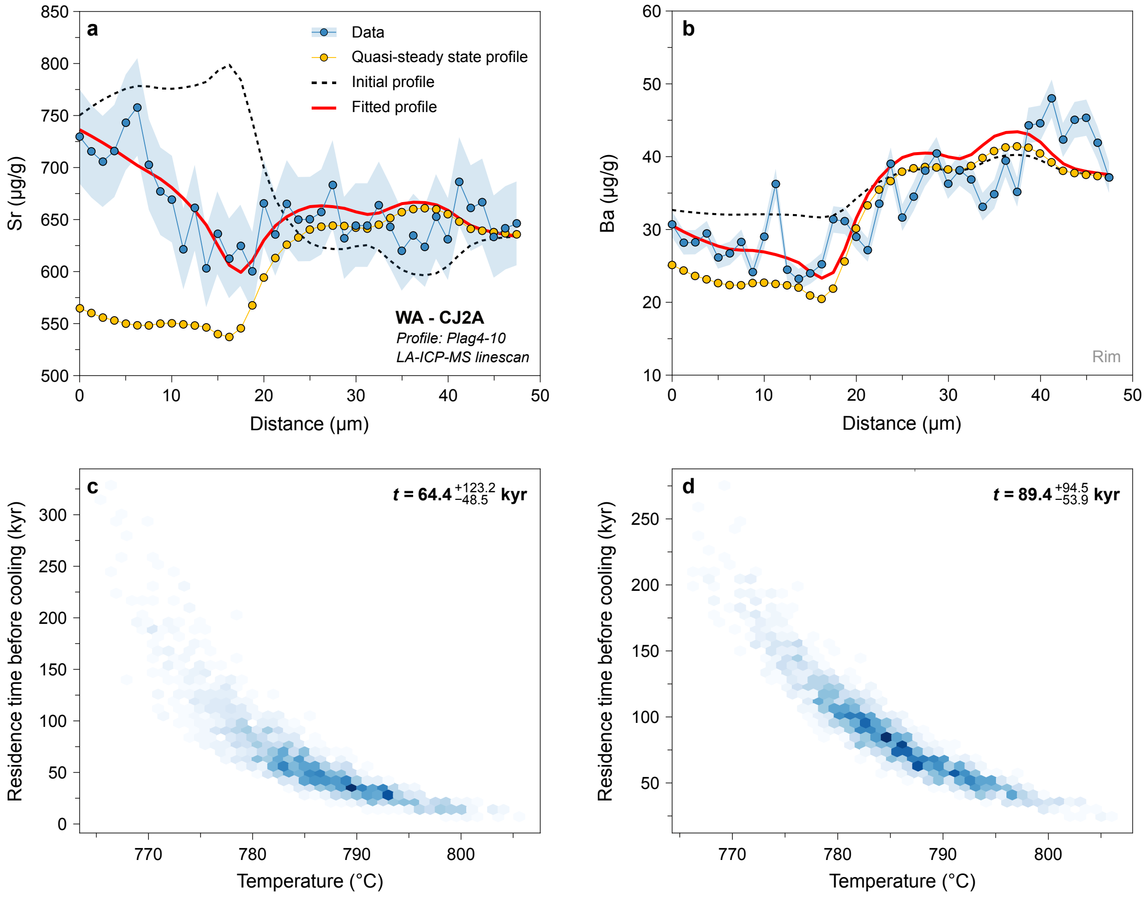
a
Sr (μg/g)
Distance (μm)
Data
Quasi-steady state profile
Initial profile
Fitted profile
WA - CJ2A
Profile: Plag4-10
LA-ICP-MS linescan
b
Ba (μg/g)
Rim
c
Residence time before cooling (kyr)
Temperature (°C)
$t = 64.4^{+123.2}_{-48.5}$ kyr
d
$t = 89.4^{+94.5}_{-53.9}$ kyr

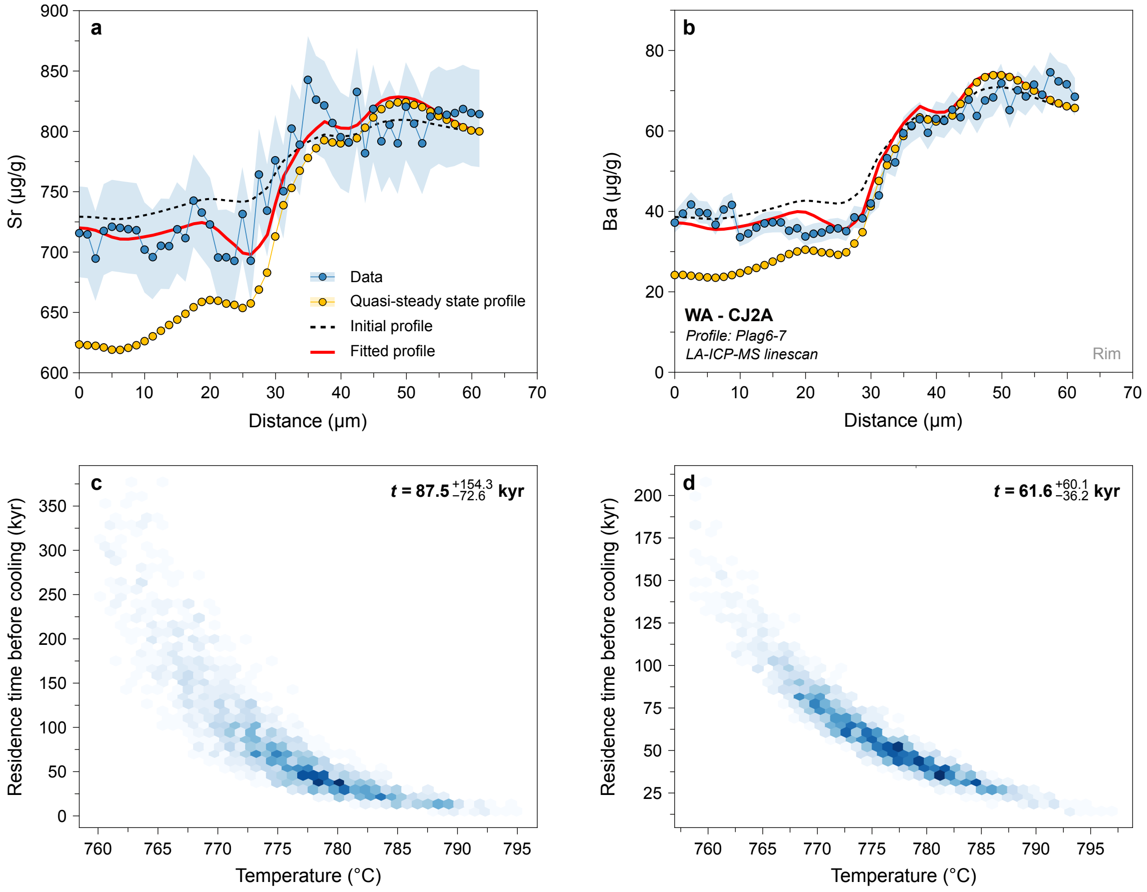
a
Sr (µg/g)
Data
Quasi-steady state profile
Initial profile
Fitted profile
Distance (µm)
b
Ba (µg/g)
WA - CJ2A
Profile: Plag6-7
LA-ICP-MS linescan
Rim
Distance (µm)
c
t = 87.5 $^{+154.3}_{-72.6}$ kyr
Residence time before cooling (kyr)
Temperature (°C)
d
t = 61.6 $^{+60.1}_{-36.2}$ kyr
Residence time before cooling (kyr)
Temperature (°C)

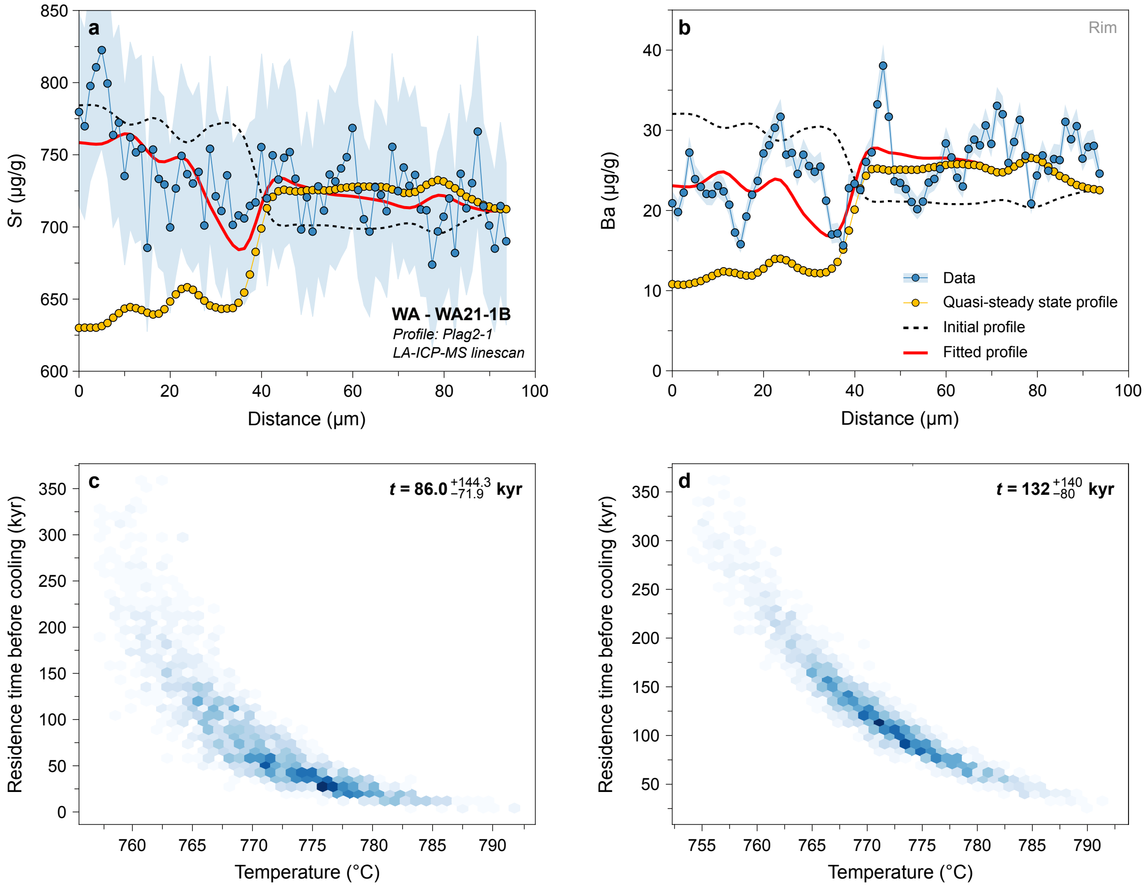
a
Sr (µg/g)
WA - WA21-1B
Profile: Plag2-1
LA-ICP-MS linescan
Distance (µm)
b
Rim
Ba (µg/g)
Data
Quasi-steady state profile
Initial profile
Fitted profile
Distance (µm)
c
t = 86.0 $^{+144.3}_{-71.9}$ kyr
Residence time before cooling (kyr)
Temperature (°C)
d
t = 132 $^{+140}_{-80}$ kyr
Residence time before cooling (kyr)
Temperature (°C)

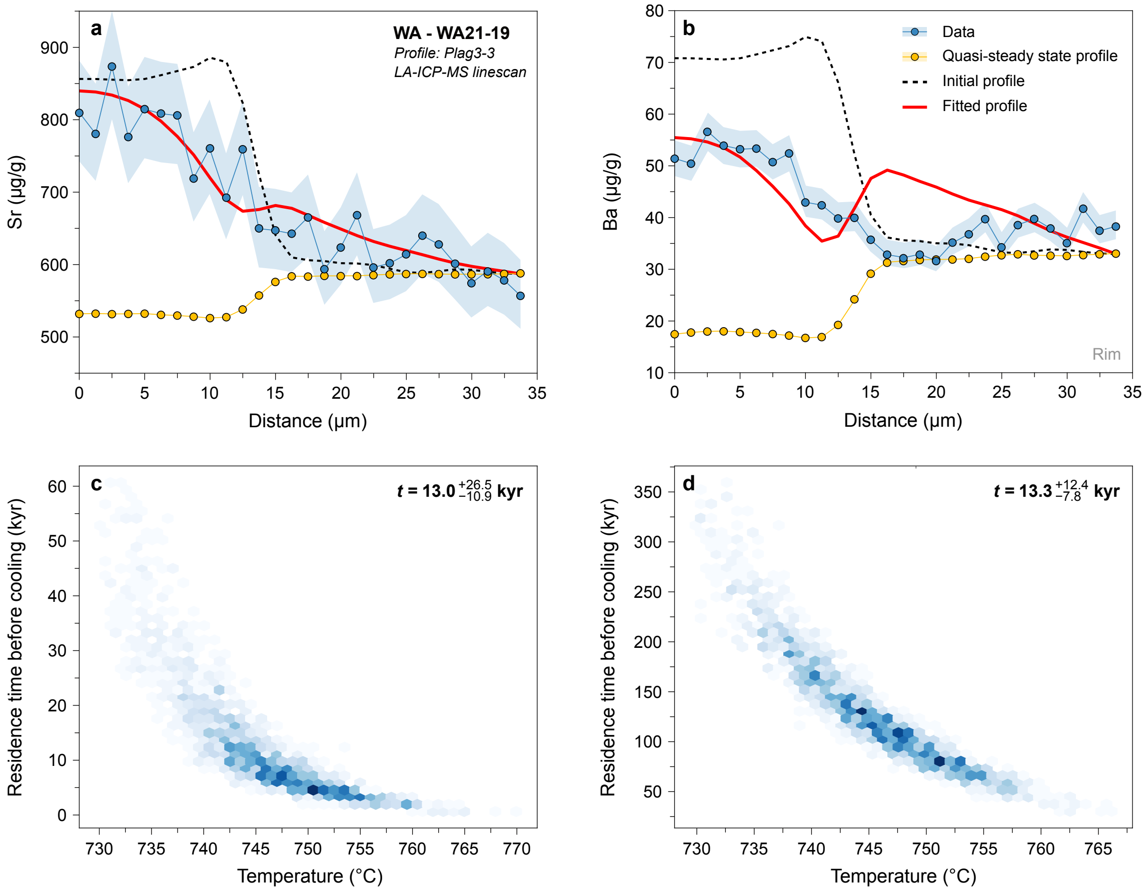
a
WA - WA21-19
Profile: Plag3-3
LA-ICP-MS linescan
Sr (µg/g)
Distance (µm)
b
Data
Quasi-steady state profile
Initial profile
Fitted profile
Ba (µg/g)
Rim
Distance (µm)
c
t = 13.0 +26.5 −10.9 kyr
Residence time before cooling (kyr)
Temperature (°C)
d
t = 13.3 +12.4 −7.8 kyr
Residence time before cooling (kyr)
Temperature (°C)

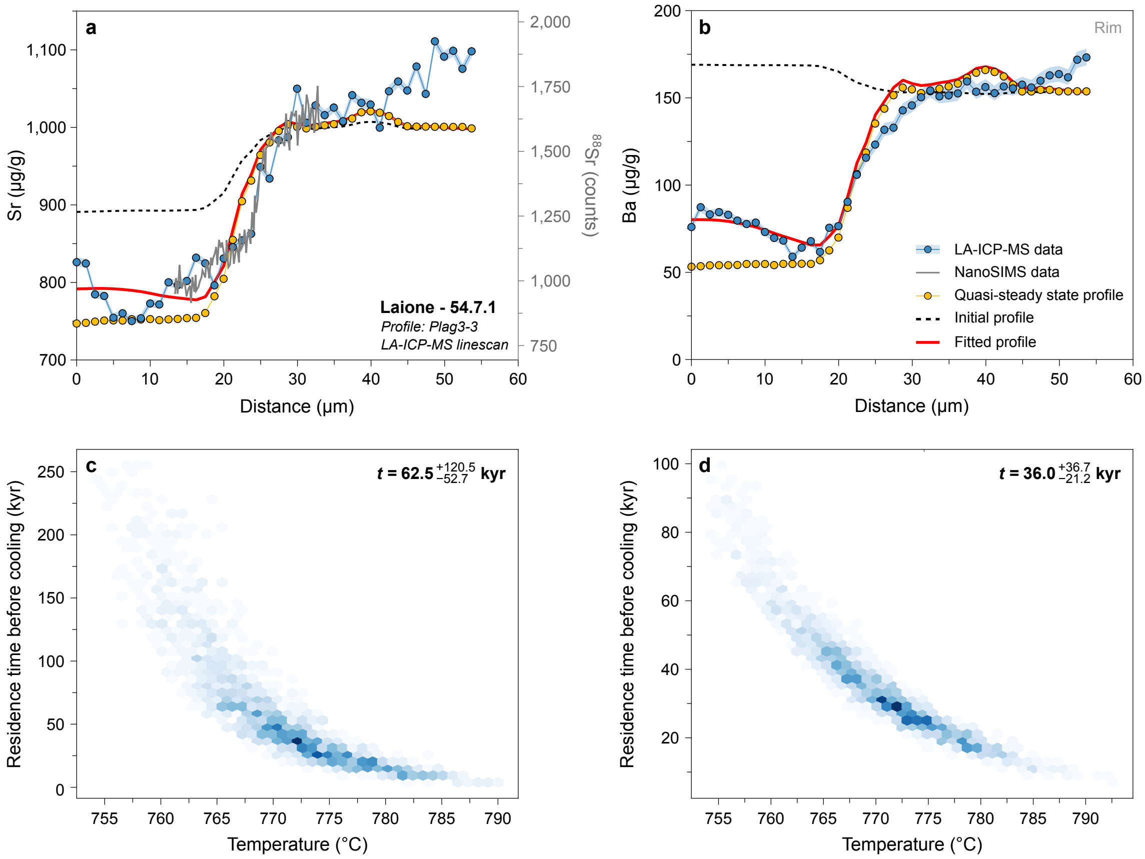
a
Sr (µg/g)
88Sr (counts)
Laione - 54.7.1
Profile: Plag3-3
LA-ICP-MS linescan
Distance (µm)
b
Rim
Ba (µg/g)
LA-ICP-MS data
NanoSIMS data
Quasi-steady state profile
Initial profile
Fitted profile
Distance (µm)
c
t = 62.5 +120.5 −52.7 kyr
Residence time before cooling (kyr)
Temperature (°C)
d
t = 36.0 +36.7 −21.2 kyr
Residence time before cooling (kyr)
Temperature (°C)

Supplementary Figure 7. Thermal model reproducing the emplacement of the Western Adamello tonalite. **a**, Model setup for the horizontal stacking of 20-m-thick vertical dikes aimed at reproducing the emplacement of the Western Adamello tonalite. The temperature of the emplaced magma (930 °C) is maintained as long as magma is flowing. After a 60-yr flow time, the system cools down for 3,000 yr until the next magma recharge. **b**, Temperature evolution of a point located at 3 km from the first dike (i.e., from the southeastern contact with the sediments) represented as temperature (°C) vs. time (yr). The solidus temperature (Johannes and Holtz, 2012; Marxer and Ulmer, 2019; Piwinskii, 1968) and modelled diffusion timescales (Fig. 12 from the main text) are shown for comparison. Note the good agreement between the plagioclase residence times inferred using diffusion modelling and the magma residence time above solidus ($\sim 10^5$ yr) inferred from thermal modelling.

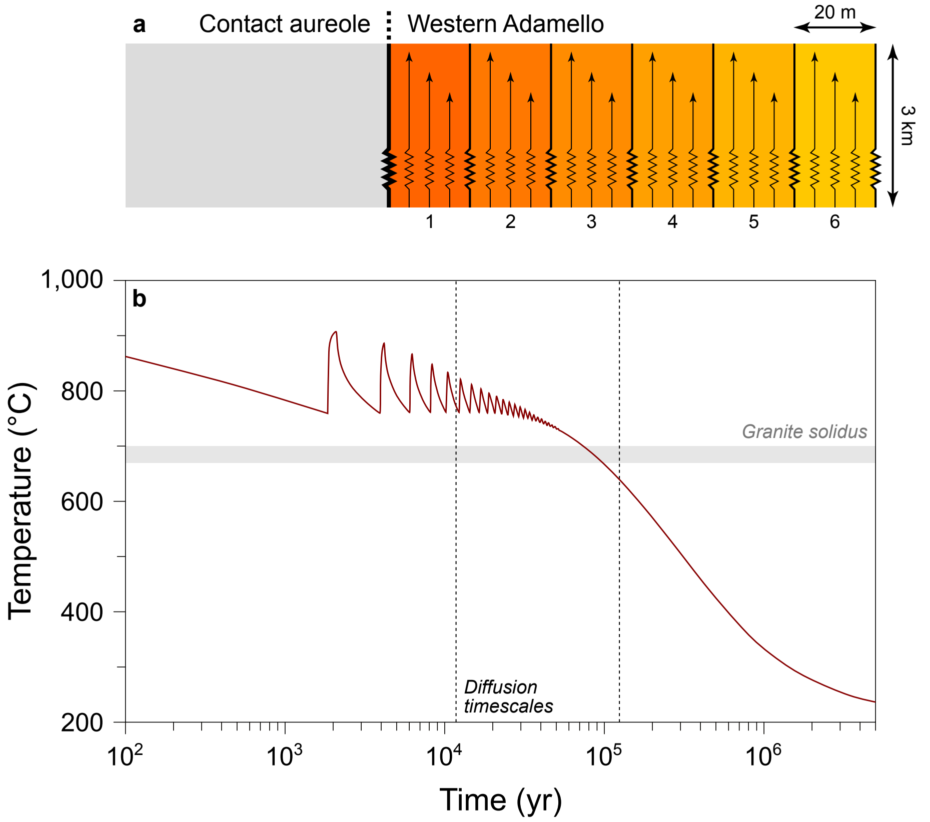

Bibliography

Cherniak, D. J. (2002). Ba diffusion in feldspar. *Geochimica et Cosmochimica Acta* 66(9), 1641–1650.

Cherniak, D. J. and Watson, E. B. (1992). A study of strontium diffusion in K-feldspar, Na-K feldspar and anorthite using Rutherford Backscattering Spectroscopy. *Earth and Planetary Science Letters* 113(3), 411–425.

Cherniak, D. J. and Watson, E. B. (1994). A study of strontium diffusion in plagioclase using Rutherford backscattering spectroscopy. *Geochimica et Cosmochimica Acta* 58(23), 5179–5190.

Costa, F., Dohmen, R. and Chakraborty, S. (2008). Time scales of magmatic processes from modeling the zoning patterns of crystals. *Reviews in Mineralogy and Geochemistry* 69(1), 545–594.

Floess, D. (2013). *Contact metamorphism and emplacement of the Western Adamello tonalite*. Ph.D. thesis, University of Lausanne.

Floess, D. and Baumgartner, L. P. (2015). Constraining magmatic fluxes through thermal modelling of contact metamorphism. *Geological Society, London, Special Publications* 422(1), 41–56.

Giletti, B. and Casserly, J. (1994). Strontium diffusion kinetics in plagioclase feldspars. *Geochimica et Cosmochimica Acta* 58(18), 3785–3793.

Grocolas, T., Bloch, E. M., Bouvier, A.-S. and Müntener, O. (2025). Diffusion of Sr and Ba in plagioclase: Composition and silica activity dependencies, and application to volcanic rocks. *Earth and Planetary Science Letters* 651, 119141.

Jochum, K. P., Weis, U., Stoll, B., Kuzmin, D., Yang, Q., Raczek, I., Jacob, D. E., Stracke, A., Birbaum, K., Frick, D. A. et al. (2011). Determination of reference values for NIST SRM 610–617 glasses following ISO guidelines. *Geostandards and Geoanalytical Research* 35(4), 397–429.

Johannes, W. and Holtz, F. (2012). *Petrogenesis and experimental petrology of granitic rocks*, volume 22. Springer Science & Business Media.

Marxer, F. and Ulmer, P. (2019). Crystallisation and zircon saturation of calc-alkaline tonalite from the Adamello Batholith at upper crustal conditions: an experimental study. *Contributions to Mineralogy and Petrology* 174(10), 84.

Middlemost, E. A. (1994). Naming materials in the magma/igneous rock system. *Earth-Science Reviews* 37(3-4), 215–224.

Müntener, O., Ulmer, P. and Blundy, J. D. (2021). Superhydrous arc magmas in the Alpine context. *Elements* 17(1), 35–40.

Mutch, E. J., Maclennan, J., Shorttle, O., Rudge, J. F. and Neave, D. A. (2021). DFENS: diffusion chronometry using finite elements and nested sampling. *Geochemistry, Geophysics, Geosystems* 22(4), e2020GC009303.

Piwinskii, A. J. (1968). Experimental studies of igneous rock series central Sierra Nevada batholith, California. *The Journal of Geology* 76(5), 548–570.

Schaltegger, U., Nowak, A., Ulianov, A., Fisher, C. M., Gerdes, A., Spikings, R., Whitehouse, M. J., Bindeman, I., Hanchar, J. M., Duff, J., Vervoort, J. D., Sheldrake, T., Caricchi, L., Brack, P. and Müntener, O. (2019). Zircon petrochronology and $^{40}Ar/^{39}Ar$ thermochronology of the Adamello Intrusive Suite, N. Italy: Monitoring the growth and decay of an incrementally assembled magmatic system. *Journal of Petrology* 60(4), 701–722.

Wu, L.-G., Li, Y., Jollands, M. C., Vermeesch, P. and Li, X.-H. (2022). Diffuser: A user-friendly program for diffusion chronometry with robust uncertainty estimation. *Computers & Geosciences* 163, 105108.
